# Battery electronification: intracell actuation and thermal management

**DOI:** 10.1038/s41467-024-49389-5

**Published:** 2024-06-25

**Authors:** Ryan S. Longchamps, Shanhai Ge, Zachary J. Trdinich, Jie Liao, Chao-Yang Wang

**Affiliations:** 1 Electrochemical Engine Center (ECEC) and Department of Mechanical Engineering, The Pennsylvania State University, University Park, PA USA; 2 EC Power, State College, PA 16803 USA

**Keywords:** Batteries, Mechanical engineering, Electrical and electronic engineering

## Abstract

Electrochemical batteries – essential to vehicle electrification and renewable energy storage – have ever-present reaction interfaces that require compromise among power, energy, lifetime, and safety. Here we report a chip-in-cell battery by integrating an ultrathin foil heater and a microswitch into the layer-by-layer architecture of a battery cell to harness intracell actuation and mutual thermal management between the heat-generating switch and heat-absorbing battery materials. The result is a two-terminal, drop-in ready battery with no bulky heat sinks or heavy wiring needed for an external high-power switch. We demonstrate rapid self-heating (∼ 60 °C min^−^^1^), low energy consumption (0.138% °C^−^^1^ of battery energy), and excellent durability (> 2000 cycles) of the greatly simplified chip-in-cell structure. The battery electronification platform unveiled here opens doors to include integrated-circuit chips inside energy storage cells for sensing, control, actuating, and wireless communications such that performance, lifetime, and safety of electrochemical energy storage devices can be internally regulated.

## Abstract (web summary)

Batteries have ever-present reaction interfaces that requires compromise among power, energy, lifetime, and safety. Here, the authors report a chip-in-cell battery by integrating an ultrathin foil heater and a microswitch into the layer-by-layer architecture of a battery cell to actuate its internal states.

## Introduction

**Fig. 1:**
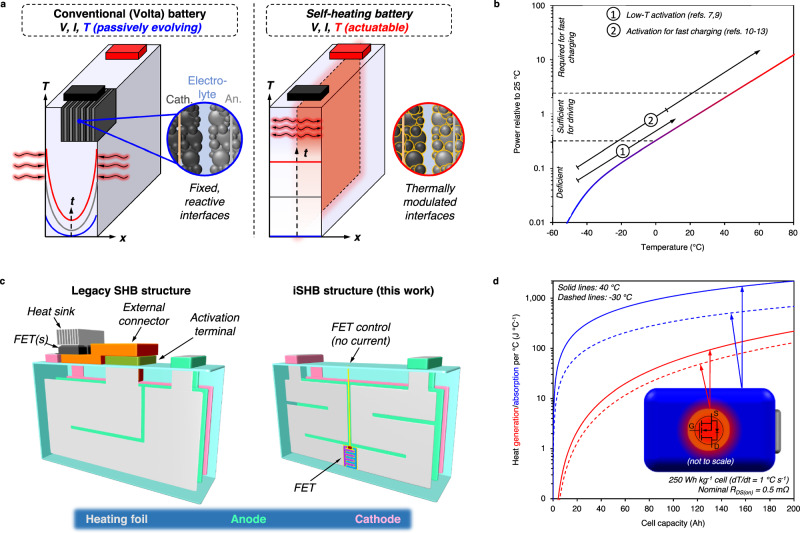
Internally actuated self-heating battery (iSHB) structure with two current-carrying terminals: concept and schematic. **a** Comparison between the conventional battery and the self-heating battery (SHB). Classical batteries are designed and operated as primarily passive devices with little control over their performance state, which depends strongly on temperature. Heating large cells necessary for electric vehicles is limited to slow external heating due to issues of spatial temperature non-uniformity. By adding an internal thermal actuator, battery temperature can be rapidly and uniformly modulated to activate the interfaces and boost power only when needed. **b** Battery power relative to that at 25 °C vs. temperature. Relative power is calculated as DCR_25°C_/DCR where DCR is the direct current resistance estimated from battery testing (Supplementary Fig. 1). The self-heating structure widens the performance window, whether it be for low-temperature performance recovery^7,9^ or enabling fast charging^10–13^. **c** Comparison between (left) the legacy SHB structure controlled by an external switch and (right) our iSHB structure based on an embedded field effect transistor (FET) switch (made by Dr. Kaiqiang Qin). **d** Heat capacity of a cell per °C temperature rise vs. cell size for 40 and −30 °C. The heat absorption capacity of a cell (250 Wh kg^−^^1^) far exceeds the heat generated by a FET of practical resistance (0.5 mΩ), making internal switching and transistor cooling feasible (see Methods for calculation). The inset of (**d**) depicts the internal FET generating heat that is dissipated to the surrounding cell, which requires heat for warming.

Alessandro Volta announced the first battery, the voltaic pile, in 1800^1^, and unveiled a battery structure that is still being used today – an anode (negative electrode) and a cathode (positive electrode) separated by an ion-conducting salt (electrolyte) often present in a porous separator that also acts as a physical barrier between the two electrodes (referred to as the “Volta battery”, herein, Fig. 1a). Subsequent battery evolution has almost exclusively relied on material modifications, i.e., changes to the electrode/electrolyte chemistry, while the Volta cell structure has remained fundamentally unchanged^2^.

Under the Volta paradigm, batteries are closed systems with no external stimuli during operation. As a result, state-of-the-art lithium-ion batteries, among others, balance power performance and aging near room temperature (RT; e.g., 15–35 °C), meaning that the battery, whether in use or at rest, maintains high power capability. Simultaneously, the interfaces between the active materials and electrolyte, which are primarily responsible for causing degradation, are perpetually reactive (Fig. 1a).

It is well established that battery performance and safety strongly hinge upon temperature. For example, lithium-ion battery (LIB) power varies three orders of magnitude from − 50 to 80 °C (Fig. 1b, Supplementary Fig. 1). Battery degradation and safety also show strong temperature dependence, with a minimum degradation rate often existing at an operating temperature and safety requiring stable response to abuse and high temperatures^3–5^. This implies that battery performance could be modulated to address a wide range of application needs while minimizing degradation and maximizing safety if the temperature could be altered on demand. However, external heating and cooling of large-format cells in present battery systems is slow (∼ 1 °C min^−^^1^) and energy-inefficient, limited by poor heat conduction in large cells and ensuing temperature non-uniformity (Fig. 1a)^6^.

Recently, a new battery coined as the “self-heating battery” (SHB) has emerged, incorporating an ultrathin internal thermal stimulator to provide safe and rapid (e.g., 60 °C min^−^^1^) battery “activation” (Fig. 1a)^7,8^. The SHB, shown in Fig.1c (left) as the legacy structure, has warranted restoration of ca. 50% nominal power and energy for 288 Wh kg^−^^1^ state-of-the-art LIBs in ultracold environments (e.g., −50 °C) rather than no performance otherwise^9^. Pre-heating with the same structure also enabled safe and healthy 10 min fast charging of energy-dense high-nickel ternary cathode-based LIBs^10–12^ and cost-effective, high-safety lithium iron phosphate (LFP)-based LIBs^13^. A similar structure was also employed to achieve practical performance of normally sluggish yet highly energy-dense all solid-state batteries with a polymer solid state electrolyte from room temperature^14^. The advent of rapid heating has also motivated a new paradigm for battery material development, favoring high temperature stability over RT rate performance^4,15–17^. Therein, temperature acts as a tool rather than the enemy, permitting batteries that are rapidly pre-heated prior to use and resting safely with minimal degradation otherwise. In this way, battery operation becomes analogous to its combustion engine counterparts, where the isolation of fuel and the energy-converting engine/turbine restricts system reactivity and instills safety. Then, only when needed, energy is released by a spark. For batteries, that spark can be the thermal energy of self-activation–the key to which is an internal actuator.

The minimal self-heating energy consumption underlies the efficacy of thermal modulation and can be calculated by:

1 $${e}_{{ACT}}=\frac{{c}_{p}}{{\eta }_{{ACT}}{{{{{\rm{SE}}}}}}}$$

where *e*_*ACT*_ is the fraction of battery energy consumed per °C of temperature rise, *c*_*p*_ is the cell specific heat, ${\eta }_{{ACT}}$ is the thermal efficiency for heating, and SE is the cell nominal specific energy. For state-of-the-art LIBs (e.g., SE ≈ 250 Wh kg^−^^1^; c_p_ ≈ 900 J kg^−^^1^ °C^−^^1^) and 100% efficiency, only 1% battery energy is required for 10 °C temperature rise! Fast heating requires rapid conversion of stored energy to heat. Thus, high current is routed from the positive terminal, through a switch, and then to the internal heating foil via a third terminal, the “activation” or “ACT” terminal in the legacy structure (Fig. 1c). For the same cell with 50 Ah capacity, 160 A is required to heat at 1 °C s^−^^1^ (see Methods section). Like the electronics that control conventional battery operation (i.e., charge-discharge), the heating control circuitry requires thermal management to subdue the temperature rise of the current-carrying switching device(s). For example, the temperature of a single field effect transistor (FET) would rise ∼ 540 °C under the above current load and adiabatic conditions (0.5 mΩ resistance; 700 J kg^−^^1^ K^−^^1^ specific heat; 1 g mass). Therefore, a major drawback of the legacy SHB structure is that large heat sinks for FET switch, bulky ACT terminal, and heavy wirings are requisite, as illustrated in Fig.1c (left).

This work presents an internally-actuated self-heating battery (iSHB) structure where FET along with ultrathin heaters are structurally and thermally fitted into the layer-by-layer architecture of a battery cell. As seen from Fig. 1c comparing the legacy structure and our iSHB structure, the iSHB structure not only achieves great simplicity in space, manufacturing, and hence cost, but also is much more amenable for fitting in layer-by-layer battery architecture. Moreover, the mutual cooling and heating needs of microelectronics and battery materials are naturally realized by placing the FET switch inside the cell, thereby containing all heat in the cell enclosure and utilizing the battery materials for heat sinking without needing the bulky ACT terminal and a giant heat sink. In Fig. 1d, the comparison of the heat generation of a FET with heat absorption capacity of a cell shows the latter exceeds the former by an order of magnitude and supports pairing them for net-zero thermal integration.

The iSHB structure possesses all functions of energy storage, power generation, and intracell temperature control. Such a cell is able to self-modulate the internal states and hence its electrode-electrolyte interfaces to regulate performance, as needed, through a wired voltage source with no current or a wireless signal. While smart batteries–which refer to those internally sensing temperature, current distribution, pressure, strain and/or stress – seek to determine battery states^18–24^, the iSHB aims to transform battery internal states in tens of seconds.

## Results

### Mutual thermal management

Figure 1c and Supplementary Fig. 2 provide schematic and electrical details of the iSHB structure. A 25 μm-thick Ni foil heating element is placed in series with a FET mounted on a thin circuit board. The heating sheet has a laminate structure, as shown in Supplementary Fig. 3a, with various layers for heat spreading, electrical insulation, and planar geometry. A conformal and chemically-inert parylene-C coating isolates a the FET and ancillary circuitry from the chemically aggressive electrolyte (see Methods and Supplementary Fig. 4). The heating sheet is inserted into the electrode stack between two anodes, and the two terminals of the heating sheet are welded to the positive and negative current collectors inside the cell, respectively. A small nickel foil lead is routed external to the cell pouch to provide voltage control of the FET. This structure removes the need for bulky ACT terminal and external wires, greatly simplifying integration into existing systems without drastic alterations. To initiate heating, the FET is turned on by applying a gate-to-source voltage (*V*_*GS*_) greater than the threshold voltage (*V*_*th*_) required to activate the conducting channel in the FET. Otherwise, the iSHB operates as a conventional electrochemical energy storage cell.

**Fig. 2:**
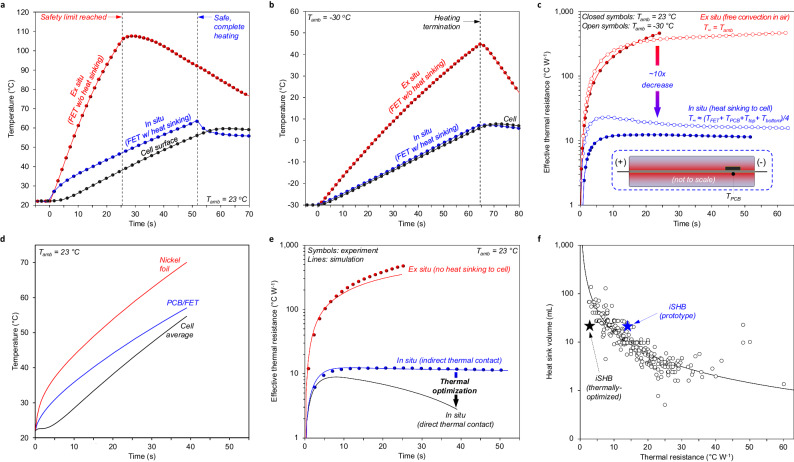
Transistor-to-cell cooling efficacy. **a**, **b** FET and PCB temperature evolution during in situ and ex situ operation for heating from 23 °C to 60 °C and − 30 °C to 5 °C, respectively. In situ, the large thermal sink of the cell prevents high FET temperature, which otherwise would rise rapidly toward safety limits. **c** Effective thermal resistance between the PCB/FET and thermal sink (ambient air for ex situ and cell average temperature for in situ). Here, *T*_*∞*_ represents the effective heat sink temperature. *T*_*FET*_ and *T*_*PCB*_ represent the top and bottom heating sheet surface temperatures at the location of the FET, respectively. *T*_*top*_ and *T*_*bottom*_ represent the top and bottom cell surface temperatures in the center of the cell. The inset illustrates the heating sheet embedded in the center of the cell, where *T*_*PCB*_ is measured on the underside of the FET. See the Methods section for analysis details. Embedding the FET in the cell provides an order of magnitude reduction in thermal resistance to enable rapid and mutual thermal management for both the cell and FET. **d** Simulated temperature evolution during heating from 23 to 60 °C for a thermally optimized iSHB where the FET is in direct thermal contact with battery materials. **e** Effective thermal resistance for the cases in **a** and **d**. The simulation suggests direct thermal contact between the FET and battery materials can achieve an additional ten-fold reduction in thermal resistance. **f** Heat sink volume vs. thermal resistance off-the-shelf heat sinks that suit one of the most common power FET packages (TO-220) available from the two of the largest electronics distributors. The prototype iSHB cooling performance is comparable to these commercial circuit board FET heat sinks with a similar volume, and the simulation suggests even higher power dissipation is possible for the same allowable FET-to-cell temperature difference. Thus, using the battery as the heat sink could roughly halve the total system volume otherwise.

For this study, we fabricated two cell types using common LIB electrode materials in a conventional electrolyte: (1) standard two-terminal cells consisting of two half-thickness pouch cells of 1.6-Ah capacity each, and (2) iSHBs of 3.2 Ah capacity (see Methods, Supplementary Table 1 and Supplementary Table 2). The half-thickness cells were used to represent conventional two-terminal cells and were also assembled into a “mock iSHB” configuration where the FET-integrated heating element was sandwiched between the twin cells to provide easy access for temperature sensing during “ex situ” and “in situ” experiments. The mock iSHB and fully-integrated iSHB configurations are illustrated in Supplementary Fig. 3b, c, d, and e. Figure 2a, b show the temperature evolution of the top and bottom heating sheets at the FET/PCB location during in situ and ex situ activation in RT and − 30 °C environments. The cell voltage evolution observed during in situ tests was imposed on the heating sheet without the cells present for ex situ tests to apply comparable heating power (Supplementary Fig. 5). Starting at RT without heat sinking to the cell, the PCB reaches ∼ 115 °C in only half the time required for in situ activation, as opposed to maintaining temperature within 11 °C of the average cell surface with heat dissipation to the battery materials. Note the FET junction temperature is estimated to stay within 0.5 °C of the PCB temperature during ex situ and in situ tests, thus representing the FET temperature well (Supplementary Fig. 6). The drastic cooling effect is also observed when heating from − 30 °C and is quantified by estimating the effective thermal resistance between the FET and the heat sink, i.e., ambient air for ex situ and the cell for in situ (Fig. 2c). The intimate contact between the FET and battery materials yields an order-of-magnitude reduction in thermal resistance (Fig. 2c).

Maximum achievable FET-to-cell cooling is then determined with a thermally optimized iSHB where all non-critical materials are eliminated from the mock iSHB, placing the FET in direct thermal contact with battery materials (Supplementary Fig. 7). A finite element model was developed to simulate the thermal response during self-heating and validated to the experimental results for the in situ and ex situ experiments at RT. See Methods and Supplementary Fig. 7 for simulation details and Supplementary Table 3, Fig. 2e, and Supplementary Figs. 8, 9 for validation results. Traces for the thermally-optimized case are shown in Fig. 2d and Supplementary Fig. 10. Analysis of the temperature evolution in the thermally optimized case suggests placing the FET in direct thermal contact with battery materials yields a further reduction in thermal resistance, approaching two orders of magnitude lower than ex situ operation and effectively constraining the switch temperature to that of the battery (Fig. 2e). This enables confident approximation of switch temperature by simply monitoring cell surface temperature.

Figure 2f and Supplementary Fig. 11 present survey results for over 400 conventional device-level heat sinks. The trend in volume vs. thermal resistance (i.e., solid line in Fig. 2f) suggests that a heat sink with ∼ 10 mL volume would be required to achieve the same thermal performance as the mock iSHB (∼ 14 °C W^−^^1^; 27 mL). Adding said 10 mL heat sink to a conventional LIB of equivalent capacity (20.6 mL; Supplementary Table 1) would yield a total system volume of ∼ 31 mL. Thus, the mock iSHB achieves a 12% reduction in system volume. For the thermally-optimized case, which more closely estimates mature iSHB implementation, simulations suggest the thermal performance can reach ∼ 5 °C W^−^^1^. A ∼ 46 mL heat sink would be required to achieve heat dissipation parity in the legacy configuration, resulting in a total system volume of ∼ 67 mL. When compared to the optimal iSHB volume (21 mL), a 68% reduction in system volume is anticipated. Note that these estimates do not consider the external conductor and ACT terminal volume, which would further increase the volume of the legacy structure and bolster the argument for the iSHB. Moreover, the improved heat dissipation of a thermally-optimized iSHB indicates an acceptable FET-to-cell temperature difference can be maintained under even higher current heating (i.e., faster heating). Of equal importance, adoption of the iSHB structure has minimal impact on the specific energy when compared to its baseline counterpart. The thermally-optimized iSHB is estimated to retain 98% of its baseline specific energy (Supplementary Table 1). The same materials used in a 50 Ah conventional cell could achieve 265 Wh kg^-1^ (ref. ^11^). Therefore, it is estimated that a 50 Ah iSHB would achieve 260 Wh kg^−^^1^, which remains state of the art for this metric.

While limiting transistor operating temperature is critical for switch lifetime, the degradation of battery materials is also temperature dependent^3–5^. Thus, ensuring in-plane temperature uniformity during heating is important for iSHB durability. To this end, the ex situ test at RT was repeated with Infrared (IR) thermographic scans of the surface for ∼15 s (Supplementary Movie 1). The thermographs in Supplementary Fig. 12 and temperature distributions in Supplementary Fig. 13 indicate that the maximum temperature variation on either the front or back surfaces was ∼ 20 °C, the magnitude of which would be suppressed in situ due to high in-plane thermal conductivity of the heat sink (i.e., cell). IR scans of the iSHB surface also show moderate in-plane non-uniformity, with a maximum surface temperature variation of ∼ 13.5 °C at the end of heating (Supplementary Fig. 14 and Supplementary Movie 2). The short duration of self-heating compared to the time scales of temperature-induced degradation (months to years) and the rapid equilibration following heating termination support the inconsequence of these levels of non-uniformity.

### Rapid and efficient thermal modulation

**Fig. 3:**
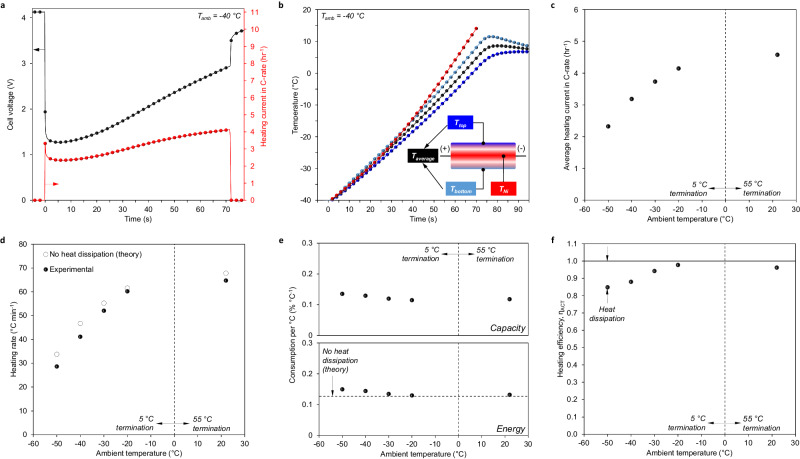
iSHB heating performance. **a** Cell voltage and heating current evolution during heating from − 40 °C to a cutoff temperature of 5 °C. **b** Temperature evolution of nickel foil (*T*_*Ni*_), top cell surface (*T*_*top*_), bottom cell surface (*T*_*bottom*_), and average cell surface (*T*_*average*_) during heating from − 40 °C. The inset illustrates the location of thermocouples (*T*_*top*_ and *T*_*bottom*_) and the heating element (*T*_*Ni*_), where the average data plotted consists of only *T*_*top*_ and *T*_*bottom*_. **c**, **d**, **e** and **f** Respectively, average discharge heating current in C-rate, theoretical and experimental heating rates, energy/capacity consumption per °C of heating, and heating efficiency vs. ambient temperature. The theoretical heating rate is calculated with Eq. 3 based on the experimental heating current while the theoretical heating energy consumption is calculated by Eq. 1. The heating efficiency in **f** is determined by the ratio of theoretical to experimental energy consumption, as shown in Eq. 22 in the Methods section.

After ex situ and in situ experiments using the mock iSHB, the final iSHB cell was constructed for extensive self-heating characterization. The performance enhancement of self-heating (i.e., the benefits to LIB performance after successful heating) has been thoroughly explored elsewhere^7–13^. Thus, we limit the performance evaluation in this work to that of self-heating at various ambient temperatures along with durability/lifetime. Figure 3 summarizes the self-heating performance of the iSHB, where the voltage, current and temperature evolution for an exemplary case of −40 °C ambient are shown. Data for other self-heating cases in −50 °C, −30 °C, −20 °C, and RT ambient are provided in Supplementary Fig. 15. Similar to the three-terminal legacy SHB structure, the iSHB voltage drops in the initial stage of heating and recovers with the swift rise in cell temperature (Fig. 3a); thus, cell resistance is reduced dramatically, and the power capability soars correspondingly. The fit of the DCR data in Supplementary Fig. 1 is used to estimate the impact of heating on cell resistance and power performance. For ambient temperatures of −50, −40, −30, and −20 °C, the cells were heated to ∼ 10 °C in all cases. This corresponds to a 98%, 93%, 85%, and 75% reduction in cell resistance, respectively. Based on the ratio of final DCR and initial DCR (before heating), thermal modulation from said low temperatures enhances power performance 42 x, 14 x, 7 x, and 4 x, respectively. During heating, the average heater temperature – as measured by utilizing the linear variation of heater resistance with temperature – is found to be within ∼ 20 °C of the cell surface average for all cases (Fig. 3b and Supplementary Fig. 15b, d, f, h). Note that design optimization of the heating structure would permit lower through-plane temperature non-uniformity by way of decreased Ni-to-cell thermal resistance. Through-plane temperature asymmetry is also observed in the iSHB, which is measured as the temperature difference between the top and bottom cell surfaces and is at most 10 °C amongst all cases (Fig. 3b). This effect would also be drastically reduced or eliminated by optimizing heater design for symmetric thermal resistance between the heater and battery materials, as in the simulated optimal case.

The high-rate discharge during heating, which ranges from ca. 2 C to 5 C on average, enables rapid heating speeds ranging from ca. 30 °C min^−^^1^ to 60 °C min^−^^1^, depending primarily on the ambient temperature and thus, the average cell temperature throughout activation (Fig. 3c, d). In contrast, external thermal management gives rise to a heating rate of ∼1 °C/min, almost two orders of magnitude slower. The theoretical iSHB heating rates in Fig. 3d, which are based on the experimental discharge current in Fig. 3c, match closely with the experiments, suggesting highly efficient heating. The capacity and energy consumption is minimal, on average consuming only 0.123 and 0.138 % °C^−^^1^, respectively (Fig. 3e). When compared to the theoretical energy consumption (0.127 % °C^−^^1^ as shown in Fig. 3e), the experimental iSHB shows heating efficiencies ranging from 85 to 98% (Fig. 3f)! Our previous study on three-terminal self-heating batteries of comparable energy density and capacity estimated heating efficiencies ranging from 82 to 93%^9^, indicating that iSHB efficiency is similar to or better than the three-terminal legacy structure while reducing total system size by more than 50%. The better result stems from the fact that the only source of heat loss in the iSHB is dissipation to the surroundings, as opposed to the additional losses to the ambient in the external heating circuit of the legacy self-heating structure, which was estimated as 3.0–5.2%^13^.

### Stable performance for long life

**Fig. 4:**
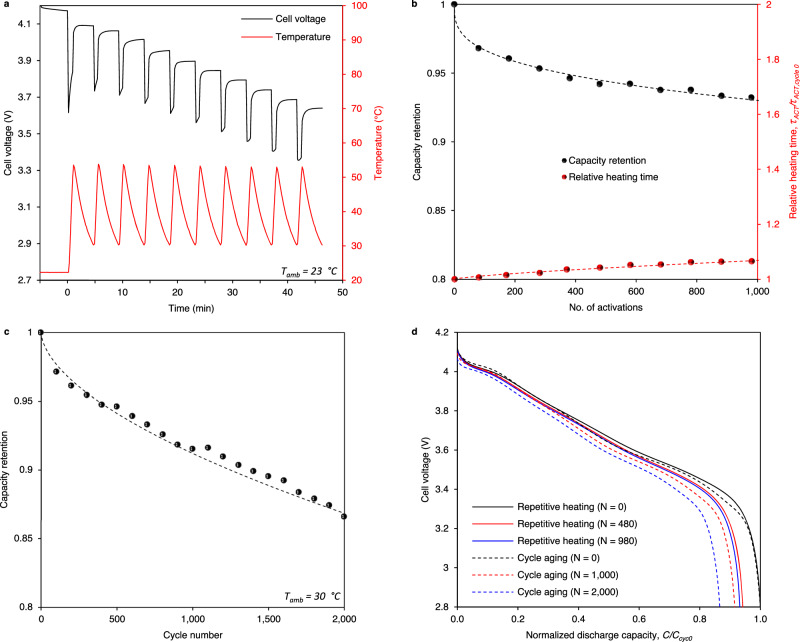
iSHB robustness: thermal and electrochemical cycling. **a** Cell voltage and temperature evolution during 10 repetitive heating cycles (activations) before the cell is recharged. **b** Capacity retention and heating time (*τ*_*ACT*_) normalized to that at cycle 0 vs. the number of activation cycles. 1000 heating cycles induces moderate capacity fade (∼ 7%) along with a degradation rate corresponding to ∼ 7% increase in heating time. **c** Capacity retention vs. cycle number during electrochemical cycle aging (charge and discharge with no heating). **d** Cell voltage vs. discharge capacity (*C*) normalized to that at cycle 0 for repetitive heating and cycle aging tests. Here, *N* represents the cycle number after which the capacity check was performed. Whether discharge is achieved conventionally or through self-heating, the rate of degradation is moderate, and no distinct abnormalities are observed during discharge.

iSHB robustness is also critical as state-of-the-art battery lifetime is on the order of decades, requiring thousands of heating events. As such, the iSHB was subjected to 1000 thermal cycles between 30 °C and ∼ 55 °C in a RT environment, following the routine shown in Fig. 4a. Despite the aggressive thermal cycling, only 7% of capacity was lost over these 1000 thermal activations. Additionally, the heating time only increased 7%, following the slow and mild increases in cell impedance (Supplementary Fig. 16). In real cases of thermally activated fast-charging cycles, this 7% fatigue due to activation and thermal cycling will be added to electrochemical degradation of the cell. Assuming the total degradation no greater than 20% and based on an estimate of 200–300 mile cruise range per fast-charging cycle enabled by each thermal activation, 1000 thermal activations thus correspond to 200–300 thousand miles of lifetime. After completing 1000 thermal activations, the cell was disassembled, and samples of the anodes neighboring either side of the heating element were extracted from two regions: FET-adjacent and Ni foil-adjacent. X-ray photoelectron spectroscopy of the graphite surface shows no notable discrepancies in the surface chemistry among the four samples (Supplementary Fig. 17). SEM micrographs also indicate no obvious disproportionate changes in microstructure (Supplementary Fig. 18). The health of the FET and the protective parylene coating is also of interest for iSHB durability, considering the corrosion potential in the presence of the electrolyte. Supplementary Fig. 19 displays optical micrographs of the FET surface without parylene and with parylene and no electrolyte exposure as well as the FET extracted after 1000 heating cycles. A comparison of the nearly identical coated surfaces along with the successful and stable operation throughout cycling shows qualitative proof of electronic device stability inside the electrochemical cell. Thermal cycling between subzero and room temperatures has been reported in refs. ^7,12^.

No interference of FET and heater integration with electrochemical performance and stability of battery materials is also evidenced in conventional cell operation. Constant current discharge rate performance tests suggest the iSHB structure has no notable effect on conventional performance (Supplementary Fig. 20). Additionally, the iSHB was subjected to 2000 standard (dis)charge cycles at 30 °C, after which it retained 86.6% of its original capacity. For context, consider an electric vehicle with 300-mile range. These 2000 cycles with an average capacity retention of 92.0% correspond to 1841 equivalent full cycles, or ∼ 890,000 km (∼ 550,000 miles) of lifetime. Extrapolation to the standard end-of-life criteria, 80% capacity retention, implies well over 1,000,000 km of lifetime.

## Discussion

The iSHB demonstrated here offers a path to achieve thermally, temporally, spatially, and gravimetrically efficient transformation of battery performance by reimagining the conventional Volta structure of the last ∼ 220 years. We term this approach “battery electronification”, generally referring to the integration of electronic components inside a battery cell to form an actively actuatable device. Broadly, breaking away from the passive, closed-system battery architecture presents an opportunity for chemistry-agnostic performance enhancement of batteries via the expansion of sensing and actuation capabilities. Such a transformation occurred in the evolution of internal combustion engines that now utilize tens or hundreds of sensors and actuators to monitor and alter the engine state, throttling performance on demand.

Batteries of current and future chemistries can morph into controllable, modulatory devices in which microelectronics and electrochemical energy storage are combined to yield unprecedented discoveries. We anticipate two areas of breakthrough progress enabled by the iSHB structure. First, achieving high-temperature stability/safety and low-temperature power simultaneously for electric vehicle batteries has been a longstanding challenge with Volta structure cells relying passively on material properties. With actuatable iSHB cells, however, one can employ materials with sluggish kinetics but high stability, such as very viscous, nonvolatile electrolytes and highly passivated or low surface area active materials that provide high-temperature safety and long life, as elegantly shown by^5,13,15–17^. Then, low-temperature power restoration is left to the iSHB self-heating structure. Also, with the advent of iSHB-enabled batteries operated at elevated temperatures, there is no more need for liquid cooling, giving rise to highly reliable and safe battery packs with no liquid passages, no pump, and no coolant leakage^11,15^.

Second, an exciting opportunity enabled by the iSHB structure is the significant improvement of calendar and cycle life of all solid-state lithium metal batteries designed to operate at elevated temperatures (e.g., 60–90 °C)^25–27^. While possessing highest energy density, lithium metal batteries are presently hindered by low coulombic efficiency stemming from side reactions between lithium metal and the electrolyte, which is most critical and damaging during long storage periods in electric vehicle applications. With a strategy to maintain stabilized, dormant electrochemical interfaces in storage at ambient temperatures but “wake up” to operate at elevated temperatures for power generation, the iSHB structure offers a path to extend the life of all solid-state lithium metal batteries, making these of highest energy density viable for electric trucks and heavy-duty vehicles critical for global commerce.

## Methods

### Theoretical calculations

#### Heating current calculations

This section describes the dependence of heating current on other battery parameters. First, an energy balance on a battery cell is considered as:

2 $$m{c}_{p}\frac{{dT}}{{dt}}={I}_{\!{ACT}}{V}_{\!{oc}}=\beta {C}_{{cell}}{V}_{\!{oc}}$$

where *m* is the cell mass, *c*_*p*_ is the specific heat of the cell, *T* is the cell temperature, *t* is time, *I*_*ACT*_ is the heating current, *V*_*oc*_ is the open-circuit cell voltage, $\beta$ is the heating current in C-rate, and *C*_*cell*_ is the cell nominal discharge capacity. Note that heat dissipation to the surroundings is neglected since the heating process is fast, and cells in a battery module or pack are well insulated. The cell mass can be recast as $\left(m={C}_{{cell}}{V}_{{nom}}/{{{{{\rm{SE}}}}}}\right)$, where *V*_*nom*_ is the cell nominal discharge voltage and SE is the cell nominal specific energy (nominal discharge energy per unit cell mass; *Specific energy and energy density calculations* section for details). This yields:

3 $$\beta=\left(\frac{{c}_{p}}{{{{{{\rm{SE}}}}}}}\right)\left(\frac{{V}_{\!{nom}}}{{V}_{\!{oc}}}\right)\left(\frac{{dT}}{{dt}}\right)$$

Thus, to heat a 250 Wh kg^−^^1^ cell at 1 °C s^−1^ with specific heat of 900 J kg^−^^1^ K^−^^1^, nominal voltage of 3.7 V, and open-circuit voltage of 4.2 V requires a C-rate of 3.17 h^−^^1^. For a 50 Ah cell, the heating current would be 159 A, or ∼ 160 A.

#### Cell heat absorption capacity and FET heat generation

This section describes the analysis underlying the results in Fig. 1d. The feasibility of the iSHB is first assessed by comparing the total rate heat generation of a transistor passing the requisite current for cell heating and the heat absorption capacity of surrounding battery materials in a cell of varying size. The rate of cell heat absorption capacity (${\dot{{{{{{\rm{q}}}}}}}}_{{{{{{\rm{abs}}}}}},{{{{{\rm{cell}}}}}}}$) is estimated starting from the energy balance in Eq. 4 that assumes no heat dissipation to the ambient, using the same justification discussed for Eq. 2. The heat capacities of the switch (field effect transistor (FET)) and Ni foil are also neglected due to their small mass (Supplementary Table 2).

4 $$m{c}_{p}\frac{{dT}}{{dt}}={\dot{q}}_{{gen},{cell}}+\sum {\dot{q}}_{{gen},i}$$

Here, ${\dot{{{{{{\rm{q}}}}}}}}_{{{{{{\rm{gen}}}}}},{{{{{\rm{cell}}}}}}}$ is the rate of heat generation from the cell materials (inefficiency of discharge) and ${\dot{{{{{{\rm{q}}}}}}}}_{{{{{{\rm{gen}}}}}},{{{{{\rm{i}}}}}}}$ is the rate of heat generation of all other heat sources (e.g., FET and Ni foil). To determine the total heat absorption capacity, the heat generation of all other heat sources is set equal to the cell heat absorption capacity (i.e., ${\dot{{{{{{\rm{q}}}}}}}}_{{{{{{\rm{abs}}}}}},{{{{{\rm{cell}}}}}}}=\sum {\dot{{{{{{\rm{q}}}}}}}}_{{{{{{\rm{gen}}}}}},{{{{{\rm{i}}}}}}}$), and Eq. 4 is recast as Eq. 5.

5 $${\dot{q}}_{{abs},{cell}}=m{c}_{p}\frac{{dT}}{{dt}}-{\dot{q}}_{{gen},{cell}}$$

The rate of cell heat generation can be approximated by Eq. 6.

6 $${\dot{q}}_{{gen},{cell}}={I}_{{ACT}}^{2}{R}_{{cell}}={\left({\beta }_{{ACT}}{C}_{{cell}}\right)}^{2}\frac{{R}_{{cell}}^{{\prime\prime} }{C}_{ {cell}}^{{\prime\prime} }}{{{C} }_{{cell}}}$$

where ${C}_{{cell}}$ is the cell capacity, ${R}_{{cell}}^{{\prime\prime} }$ is the area-normalized cell resistance, and ${C}_{{cell}}^{{\prime\prime} }$ is the areal capacity of the cathode (capacity per electrode footprint area). The combination of Eqs. 3, 5, 6, and $m=({C}_{{cell}}{V}_{{nom}})/{{{{{\rm{SE}}}}}}$ yields Eq. 7:

7 $${\dot{q}}_{{abs},{cell}}=\frac{{C}_{{cell}}{V}_{\!{nom}}{c}_{p}}{{{{{{\rm{SE}}}}}}}\left(\frac{{dT}}{{dt}}\right)\left[1-\frac{{c}_{p}{V}_{\!{nom}}{R}_{{cell}}^{{\prime\prime} }{C}_{ {cell}}^{{\prime\prime} }}{({SE}){V}_{\!{oc}}^{2}}\frac{{dT}}{{dt}}\right]$$

The FET heat generation is calculated as:

8 $${\dot{q}}_{{gen},{FET}}={I}_{{ACT}}^{2}{R}_{{FET}}={\left[{C}_{{cell}}\frac{{c}_{p}{V}_{\!{nom}}}{({{{{{\rm{SE}}}}}}){V}_{\!{oc}}}\left(\frac{{dT}}{{dt}}\right)\right]}^{2}{R}_{{FET}}$$

The total heat generated per °C of cell temperature rise can then be determined by dividing Eqs. 7 and 8 by (*dT/dt*), as plotted in Fig. 1d for heating at 40 °C and −30 °C for a cell with SE of 250 Wh kg^−^^1^, a heating rate of 1 °C s^−^^1^, and a FET of low but achievable resistance (e.g., 0.5 mΩ, see IPT004N03LATMA1, Infineon Technologies). Note the temperature dependence of cell resistance is determined from the DCR data in Supplementary Fig. 1, and that of the FET is approximated from that of an off-the-shelf metal oxide semiconductor FET (MOSFET; IRFP3077PBF, Infineon Technologies). At both high and low temperature, the cell possesses at least an order of magnitude greater heat absorption capacity than the heat generated by the FET, indicating that the cell thermal sinking capacity and the FET heat generation are well paired for effective mutual thermal management.

### iSHB characterization: experimental and numerical

#### Cell materials and fabrication

We fabricated 1.6 Ah conventional pouch cells, and 3.2 Ah iSHB pouch cells using Li(Ni_0.8_Co_0.1_Mn_0.1_)O_2_ (NMC811) as cathodes and graphite as anodes with 1M LiPF_6_ in ethylene carbonate/ethyl methyl carbonate (3:7 by weight) + 2% vinylene carbonate as electrolyte (Soulbrain, Michigan). The negative-to-positive capacity ratio was ∼ 1.1. All cells used a stacked electrode design. The 1.6 Ah cells had 7 anode layers and 6 cathode layers while the 3.2 Ah cells had 14 anodes and 12 cathodes. A ceramic-coated separator (Celgard 2325) of 25 μm thickness was used.

For iSHB cells, a gallium nitride field effect transistor (EPC2021, EPC Technologies, USA) was soldered onto a flexible printed circuit board (PCB; Best Technologies, Ltd., China) with 15 mm × 15 mm area and 95 μm thickness. Ni foil leads were then soldered to the PCB connections for the drain, source, and gate. The total heating sheet resistance was sized at ∼ 230 mΩ. Adhesive heat spreader with 10 μm thickness (Panasonic EYG-A121801M, Japan) was then applied to the top and bottom of the Ni-PCB assembly after a 20 μm layer of adhesive polyimide was applied to both sides of the Ni foil/PCB for electrical insulation. To create a uniform thickness approximately equal to the height of the FET, three layers of adhesive polyimide film (670 μm total thickness) were adhered to the heat spreader surface on the side of the FET. Next, the Ni foil tabs that get welded to the electrode current collectors were masked, and a parylene-C conformal coating was applied to provide a barrier for electrical insulation and anti-corrosion. Parylene-C was deposited at room temperature using a Labcoter 2 Model PDS 2010 Parylene Deposition Unit (Specialty Coating Systems, Inc). The final coating thickness was measured at ∼ 15 μm using optical microscopy (Supplementary Fig. 4). Finally, the entire heating sheet was laminated with 25 μm PET film. The heating sheet was inserted in the center of the electrode stack between two single sided anodes.

The cathodes were prepared by coating N-methylpyrrolidone-based slurry onto 13 μm Al foil, whose dry material consisted of 97.7wt.% NMC811. The anodes were prepared by coating deionized water-based slurry onto 8 μm Cu foil, whose dry weight consisted of 97.7 wt.% graphite. The mass loadings of NMC811 and graphite were 16.8 and 10.8 mg cm^−^^2^, respectively. All cells had a 110 mm × 80 mm footprint area. The conventional LIB and iSHB cells had thicknesses of 2.6 and 6.0 mm, respectively. See Supplementary Table 1 for additional details on cell design and performance metrics.

#### Specific energy and energy density calculations

The specific energies reported for the baseline LIB and iSHB prototype in Supplementary Table 1 were determined by measuring the discharge energy at a C-rate of C/3 and room temperature (23 °C ± 2 °C) and the mass of the cell. The former was then divided by the latter to calculate the specific energy. The specific energy of the optimized iSHB was estimated dividing the discharge energy of the iSHB prototype cell by the estimated mass of the optimized iSHB. The details of the optimized iSHB mass estimate are provided in Supplementary Table 2.

The energy densities reported for the baseline LIB and ISHB prototype in Supplementary Table 1 were determined by diving the discharge energy as measured above by the volume of the cell. The volume of the cell was calculated as the product of the cell thickness and the footprint of the pouch, including the flange regions where the pouch is sealed to the tabs and excluding the flange regions along the sides of the pouch, as these would be folded tightly to the side of the cell in a practical system. The energy density of the optimized iSHB was estimated by dividing the discharge energy of the prototype iSHB by the volume of the optimized iSHB. The volume of the optimized iSHB was determined by adding the volume of the optimized heating sheet (0.41 mL) to the volume of the baseline LIB (20.6 mL).

#### In situ and ex situ heating tests

All environmentally controlled tests were performed in an environmental chamber (Tenney Environmental), and charge and discharge tests were performed using a battery tester (Arbin BT-2000). All cell charging was performed at RT following the constant current-constant voltage (CC-CV) charging protocol with a charge rate of C/3, CC cutoff voltage of 4.2 V, CV cutoff current of C/20. All heating experiments (in situ and iSHB) were performed at 100% state of charge (SOC). All thermocouple temperatures and voltages (Cell or heating sheet, Ni foil, shunt) during heating tests were recorded at a sampling rate of 5 Hz using a data acquisition system (USB-2408; Measurement Computing).

For in situ heating sheet temperature measurements, the iSHB heating sheet was sandwiched between two 1.6 Ah, two-terminal, LIBs to form a “mock” iSHB (Supplementary Fig. 3b, c, d). A thin layer of silicone thermal paste was deposited between the heating element and cells and the entire stack was clamped between 3 cm-thick polyethylene foam to minimize thermal resistance between the heating sheet and the cell pouches. The two cells were wired in parallel, and a shunt resistor (0.75 mΩ, Ohmite) was connected in series between the positive cell terminal and the positive heating sheet connection for current measurement. T-type thermocouples with 0.254 mm tip diameter (Omega Engineering) were used for all temperature surface temperature measurements except the infrared thermography scans. The thermocouples were mounted on the heating sheet on the top and bottom in the center of the FET footprint. To turn on the FET, a separate Arbin channel was used to apply a gate voltage (*V*_*GS*_) of 4.5 V. In RT ambient, the cell-sheet assembly was heated to an average outer cell surface temperature of 55 °C, after which the temperature rose to ca. 60 °C. For −30 °C ambient, the cell was soaked in the environmental chamber for > 6 h. to allow for thermal equilibration. To terminate heating, an average surface temperature cutoff of 5 °C was used, after which the cell temperature rose toward ca. 10 °C.

Ex situ tests were performed in the absence of the two half-thickness cells, insulating foam, and clamping pressure. The thermocouples were placed as in the in situ tests (Supplementary Fig. 3c). The power level observed during in situ tests was emulated by fitting a piecewise function to the cell voltage trend during in situ tests and programming the Arbin for such time-dependent voltage control (Fig. 2c, d). The test was run until the time corresponded to the heating time observed during in situ tests (51.6 s for RT ambient and 64.5 s for −30 °C ambient) or until the FET or PCB temperatures reached 115 °C. In the RT test, the PCB temperature reached 115 °C in 25.2 seconds while the test in −30 °C ambient ran for the full 64.5 s without either temperature reaching the safety cutoff.

To estimate the thermal resistance between the circuit board and the heat sink (ambient air for ex situ and cell for in situ), an energy balance on the FET was applied as follows:

9 $${\left[m{c}_{p}\left(\frac{{dT}}{{dt}}\right)\right]}_{{FET}}={I}_{{ACT}}^{2}{R}_{{FET}}-\frac{({T}_{{PCB}}-{T}_{{\infty }})}{{R}_{{th},B-C}}$$

where ${m}_{{FET}}$, ${c}_{p,{FET}}$, and ${\left(\frac{{dT}}{{dt}}\right)}_{{FET}}$ are the mass, specific heat, and rate of temperature rise of the FET, respectively. ${I}_{{ACT}}$ is the heating current, ${R}_{{FET}}$ is the FET resistance, ${{{{{{\rm{T}}}}}}}_{{{{{{\rm{PCB}}}}}}}$ is the PCB temperature and is assumed to be equal to the FET temperature, ${T}_{{\infty }}$ is the effective thermal sink temperature, and ${R}_{{th},B-C}$ is the effective thermal resistance between the PCB and the ambient. ${c}_{p,{FET}}$ is estimated as shown in Supplementary Table 3 based on 50:50 vol% gallium nitride: silicon. The FET/junction temperature and rate of temperature rise are estimated from the PCB rate of temperature rise, supported by the negligible difference between the circuit board and FET junction temperature (Supplementary Fig. 6). Additionally, the FET resistance is evaluated at *T*_*PCB*_ based on the temperature dependence of resistance provided by the manufacturer. For ex situ tests, ${T}_{{\infty }}$ was the ambient temperature, and in situ tests, ${T}_{{\infty }}$ was estimated as the average of the measured FET (top), PCB (bottom), and cell surface temperatures, which assumes a linear spatial temperature distribution from the cell surface to the heating sheet surface.

#### Numerical simulation of iSHB heating

A semi-analytical, finite element, thermal model was developed in COMSOL Multiphysics simulation software to investigate the maximum heating performance of the iSHB under optimal conditions. Three-dimensional simulation is adopted since through- and in-plane effects are critical in the iSHB. To reduce computational expense, the heating sheet and cell were treated as symmetric about the plane that passes through the center of the FET along the longer of the two centerlines. Equation 10 describes the characteristic equation governing heat conduction in solids.

10 $$\rho {c}_{p}\frac{\partial T}{\partial t}={k{{{{{\boldsymbol{\nabla }}}}}}}^{2}T+{\dot{q}}^{{\prime} {\prime} {\prime} }$$

Here, $\rho$ is the material density and ${\dot{q}}^{{\prime} {\prime} {\prime} }$ is the volumetric heat generation, which is only non-zero in the Ni foil, FET, and LIB cell, when present. Two boundary conditions were applied in the model: Thermally insulated (Eq. 11) and convective (Eq. 12).

11 $${{{{{\bf{n}}}}}}\cdot {{{{{\boldsymbol{\nabla }}}}}}T=0$$

12 $$-k({{{{{\bf{n}}}}}}\cdot {{{{{\boldsymbol{\nabla }}}}}}T)=h({T}_{s}-{T}_{{amb}})$$

Here, ${{{{{\bf{n}}}}}}$ is the outward facing surface normal vector. The volumetric heat generation in the Ni foil, FET, and cell (in situ only) are defined by Eqs. 13, 14, and 15, respectively.

13 $${\dot{q}}_{{Ni}}^{{\prime} {\prime} {\prime} }=\frac{{I}_{{ACT}}^{2}{R}_{{Ni}}}{{{{{{{\mathcal{V}}}}}}}_{{Ni},{iSHB}}}$$

14 $${\dot{q}}_{{FET}}^{{\prime} {\prime} {\prime} }=\frac{{I}_{{ACT}}^{2}{R}_{{FET}}}{{{{{{{\mathcal{V}}}}}}}_{{Ni},{iSHB}}}$$

15 $${\dot{q}}_{{cell},\,i}^{{\prime} {\prime} {\prime} }=\frac{{I}_{{ACT}}^{2}{R}_{{cell},i}}{{{{{{{\mathcal{V}}}}}}}_{{cell},{iSHB}}}$$

Here, ${{{{{{\mathcal{V}}}}}}}_{i,{iSHB}}$ is the volume of the i^th^ component in the prototype iSHB. Note, the cell heat generation for the top and bottom portions were calculated and applied independently based on the respective domain-averaged temperatures.

Three cases were simulated corresponding to heating in a RT ambient environment. First, the heating sheet was simulated as in the ex situ test and compared to experimental data for model tuning/validation. Then, the in situ heating test was simulated and compared with experimental data. Finally, an optimal heating sheet was developed as a best-case scenario with no polyimide or heat spreader to achieve the most intimate geometric and thermal contact between the heater and battery. For the ex situ case, the time-dependent voltage evolution was applied across the sheet, as in the experiment, and the heating current was determined based on Eq. 16.

16 $${I}_{{ACT}}=\frac{{V}_{{cell}}}{{R}_{{Ni}}}$$

For simplicity, the FET resistance is not considered here, as it only contributes 0.7% of the total resistance. For the in situ case, the cell voltage and current were calculated by Eqs. 17 and 18, respectively.

17 $${V}_{\!{cell}}={V}_{\!{oc}}-{I}_{{ACT}} * {R}_{{cell}}$$

18 $${I}_{\!{ACT}}=\frac{{V}_{{cell}}}{{R}_{{Ni}}+{R}_{{cell}}}$$

The Ni foil, FET, and cell resistances are modeled empirically and were evaluated at their respective domain-averaged temperatures, based on Eqs. 19, 20, and 21, respectively.

19 $${R}_{{Ni}}=0.235\varOmega \left(1+\left(0.0047\,{K}^{-1}\right)\left(T-294.1K\right)\right)$$

20 $${R}_{{FET}}=0.0018\varOmega \left(1+\left(0.00973\,{K}^{-1}\right)\left(T-298K\right)\right)$$

21 $${R}_{{cell}}=f\left[0.000122{e}^{-0.1806{K}^{-1}\left(T-273.15K\right)}+0.060754{e}^{-0.0454{K}^{-1}(T-273.15K)}\right]$$

*R*_*Ni*_ was determined from experimental calibration of the heating sheet, *R*_*FET*_ was determined based on a linear fit of the temperature dependency of resistance provided by the manufacturer, and *R*_*cell*_ corresponds to the fit series in Supplementary Fig. 1. Note that the pre-factor, *f*, is applied to tune the resistance based on cell-to-cell and test rig variation, and a final value of 1.5 was applied. This is largely attributed to the many connections required in the in situ test rig and the small weld points for connection to the cell tabs, which added resistance to the heating circuit.

Supplementary Table 2 shows the material properties applied to various discrete volumes, which, in some cases represent a composite of multiple base materials. A mesh was applied to all geometries using the built-in physics-controlled mesh in COMSOL. All simulations used a time step of 0.1 s and a direct solver (PARDISO). Lastly, convection coefficients of 20 and 30 W m^−^^2^ K^−^^1^ were applied in the ex situ and in situ simulations, respectively. The ex situ experiment was performed under natural convection while the cell in the in situ experiment was surrounded by additional thermal mass (compressed foam). Given the short heating time, the outer material does not have time to reach steady state and acts as a thermal sink while its temperature evolves, justifying an increase in *h*.

#### Survey of common heat sinks

The catalogs of two of the largest electronic component distributors, Mouser Electronics and Digikey, were surveyed for heat sinks that suit the most common FET package, TO-220. The results were filtered to only include unique part numbers, and the volume of the resulting 441 heat sinks was computed based on the appropriate heat sink footprints (i.e., length*width*height).

#### Infrared thermography

Infrared thermography measurements were obtained using an ImageIR 8300 (INFRATEC) high-end thermography camera with a 50 mm telephoto lens. Prior to IR scans, the sample was painted with matte black spray paint and allowed to dry for one day. Two coats were applied for even coverage, as necessary. The test configuration was identical to prior infrared thermography of self-heating batteries in our group^15^. Prior to scans of a new sample or at a new temperature range, calibration validated by a thermocouple mounted to the imaged surface was performed. Emissivity corrections were performed based on thermocouple measurements of the sample surface at RT and 40 °C.

#### iSHB self-heating tests

The final, fully integrated iSHB (i.e., FET inside cell enclosure/pouch), was used for the experimental study of self-heating performance. The iSHB was insulated during heating tests to best emulate the insulated conditions of a cell in a battery module or pack. The iSHB heating sheet was instrumented for current measurement by soldering a 0.5 mΩ surface mount shunt resistor (CSNL2512 (thickness: 350 μm, Stackpole Electronics, Inc.) in series with the Ni foil. Two additional Ni foil leads with 2 mm width were soldered to the top pads of the resistor to measure the voltage drop across the shunt. In cell assembly, 4 mm wide Ni tabs were welded to the Ni leads and passed through the pouch (hot sealed) for external measurement. The shunt resistance was measured at 20 °C intervals from −60 °C to 60 °C and was found to only vary from the average by + 0.4%/− 0.6%, indicating stable current measurements across the wide temperature range for self-heating tests. During heating tests, the state of the FET was controlled by a separate Arbin channel, where *V*_*GS*_ = 4.5 V was applied to turn on the FET, as in the in/ex situ tests. The average Ni foil temperature was also monitored during self-heating, utilizing the following empirical resistance temperature detector (RTD) calibration: ${{{{{{\rm{R}}}}}}}_{{{{{{\rm{foil}}}}}}}=230{{{{{\rm{m}}}}}}\Omega \left[1+0.00433^{{\circ} }{{{{{\rm{C}}}}}}^{-1}\left({{{{{\rm{T}}}}}}-21.1^{{\circ} }{{{{{\rm{C}}}}}}\right)\right]$ with R^2^ = 0.998.

Heating was terminated at an average surface temperature of 55 °C for the RT ambient temperature test and 5 °C for ambient temperatures of −20, −30, −40, and −50 °C. The average cell temperature was estimated as the spatial average of the Ni foil temperature and the cell surface temperature, which assumed a linear temperature profile throughout the cell. The self-heating rate was calculated as the difference between the final and initial cell temperatures divided by the time to reach the cutoff surface temperature. Self-heating capacity consumption was calculated as the product of the average self-heating current and the self-heating time. Self-heating energy consumption was calculated as the product of the self-heating capacity and the open circuit voltage prior to self-heating since the open-circuit voltage remains approximately constant during self-heating due to low state-of-charge (SOC) change. The heating efficiency (η_ACT_) shown in Fig. 3f was calculated as follows:

22 $${\eta }_{{ACT}}=\frac{{c}_{p,{cell}}{\left({T}_{f}-{T}_{i}\right)}_{{ACT}}}{{{{{{\rm{SE}}}}}}{\left(I\tau {V}_{\!{oc}}\right)}_{{ACT}}}$$

where ${c}_{p,{cell}}$ is the respective cell specific heat (see Supplementary Table 2), *T*_*f*_ is average of the Ni foil and surface temperatures at the end of heating, *T*_*i*_ is average of the Ni foil and surface temperatures at the beginning of heating, SE is the nominal cell specific energy, *τ*_*ACT*_ is the heating time, and *V*_*oc,ACT*_ is the average open circuit voltage during heating (estimated as the initial open circuit cell voltage).

#### Repetitive thermal cycling tests

The initial capacity of the iSHB was characterized by discharging the cell from 100% SOC at a C/3 rate to 2.8 V at RT (referred to as reference performance test (RPT), hereafter). For thermal cycling, an uninsulated iSHB started at 100% SOC and RT. Heating was initiated as in the heating characterization tests (i.e., *V*_*GS*_ = 4.5 V), and proceeded until the average cell surface temperature reached 50 °C, after which it rose to ∼ 55 °C. The cell was then cooled naturally to 30 °C, and the heating process was repeated nine more times (Fig. 4a). After 10 heating cycles, the cell was recharged at RT, and the heating-charging process was repeated. After each phase of thermal cycling, an RPT was performed (Fig. 4b, d). The initial thermal cycling-RPT phase consisted of 80 thermal cycles, after which the RPT interval was adjusted to 100 thermal cycles. After 980 heating cycles, the cell was fully discharged and disassembled in a glove box (MBraun) with an Argon environment for post-mortem analysis.

#### Electrochemical cycle aging and C-rate tests

The cycle aging test was carried out at constant temperature in an oven set to 30 °C. The cycling protocol consisted of a CC charge to 4.2 V at a rate of C/2, 5 min. rest, CC discharge to 2.8 V at a rate of 1 C, and a 5 min. rest. After every 50 cycles, the test was interrupted, and a reference performance test (RPT) was performed following the C/3 CCCV charge protocol with an upper cutoff voltage of 4.2 V and a cutoff current of C/20. After resting one hour, the cell was discharged at C/3 rate to a lower cutoff voltage of 2.8 V (Fig. 4c, d). The C-rate test was performed on fresh SHB and iSHB cells at room temperature following the same charge protocol above. After resting for one hour, the cells were discharged at C/3, 1 C, and 3 C rates with a lower cutoff voltage of 2.8 V.

#### Direct current resistance characterization

For DCR measurements, a three-terminal self-heating battery was constructed with the same electrode layers and a Ni foil sized at 228 mΩ, and self-heating tests were executed with the same procedure used for the iSHB. All DCR data was approximated from the first 30 s of self-heating tests of the SHB. The representative temperature was taken as the average surface temperature during those 30 s of heating. DCR is calculated with Eq. 23 where ${A}_{{cath},{tot}}$ is the total cathode area for the respective cell, ${V}_{{cell},30s}$ is the cell voltage at thirty seconds, and ${I}_{{ACT},30s}$ is the average current during the 30 s discharge.

23 $${{{{{\rm{DCR}}}}}}={A}_{{cath},{tot}}\frac{({V}_{\!{oc}}-{V}_{\!{cell},30s})}{{I}_{{ACT},30s}}$$

#### Optical and scanning electron microscopy

Optical microscopy was performed with a Trinocular Dual-illumination Metallurgical Microscope (ME580TA-PZ-2L-18M3, AmScope). Imaging was performed in a lab environment under standard atmospheric conditions. All FETs imaged underwent the same fabrication process up to the point that distinguishes their coating/exposure status (i.e., soldered onto PCB, and all laminate layers applied). The FET exposed to electrolyte and thermally cycled 980 times was harvested in a glove box and washed with dimethyl carbonate (DMC) to remove any residual salt prior to exposure to atmospheric air to avoid any concerns of coating disruption or FET corrosion.

Scanning electron microscopy (SEM) imaging was performed by first extracting the electrode samples from the fully discharged cell after thermal cycling and washing with DMC inside an Argon-filled glovebox with < 0.1 ppm oxygen and moisture. Samples were extracted from the two anodes adjacent to the heating sheet at the regions over the FET footprint and the Ni foil halfway between the FET and the bottom heating sheet edge. SEM imaging was performed on an FEI Nova NanoSEM 630 SEM instrument.

#### X-ray photoelectron microscopy

For X-ray photoelectron spectroscopy (XPS), the samples were extracted as in SEM imaging but were washed three times with DMC to ensure the surface chemistry analysis was not skewed by the presence of Li salt (Lithium hexafluorophosphate). A PHI VersaProbe II Scanning XPS Microprobe was used for spectroscopic analysis. The samples were loaded in a glovebox and transferred into the instrument through a vacuum transfer vessel.

### Supplementary information

Supplementary Information

Peer Review File

Description of Additional Supplementary Files

Supplementary Movie 1

Supplementary Movie 2

## Data Availability

All data supporting the research in this paper are available in the main text and Supplementary Information, and source data can be obtained through reasonable requests from corresponding authors.
