# Supplementary material for: Battery electronification: intracell actuation and thermal management
Source: Nat Commun. 2024 Jun 25;15:5373. doi: 10.1038/s41467-024-49389-5 (PMC11199607; doi:10.1038/s41467-024-49389-5)
Supplement: Supplementary file 1 — Supplementary Information [file 41467_2024_49389_MOESM1_ESM.pdf]

Supplementary Information

Battery Electronification: Intracell Actuation and Thermal Management

Ryan S. Longchamps[1,2], Shanhai Ge[1], Zachary J. Trdinich[1], Jie Liao[1], Chao-Yang Wang[1]*

[1]Electrochemical Engine Center (ECEC) and Department of Mechanical Engineering, The Pennsylvania State University, University Park, PA, USA

[2]EC Power, State College, PA 16803, USA

*Corresponding author. Email: cxw31@psu.edu

Supplementary Figures

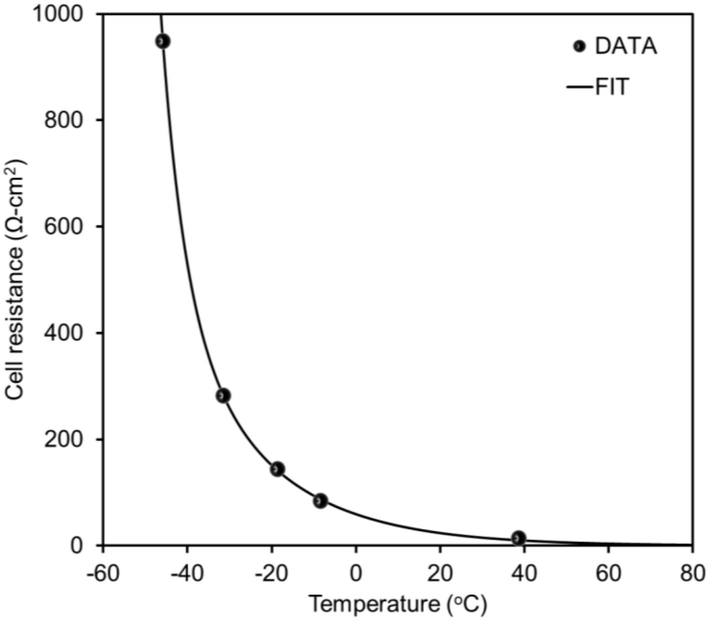

Supplementary Fig. 1 | Cell resistance vs. temperature. Direct current resistance (DCR) vs. average cell surface temperature during self-heating from -50, -40, -30, -20, and 23 °C.

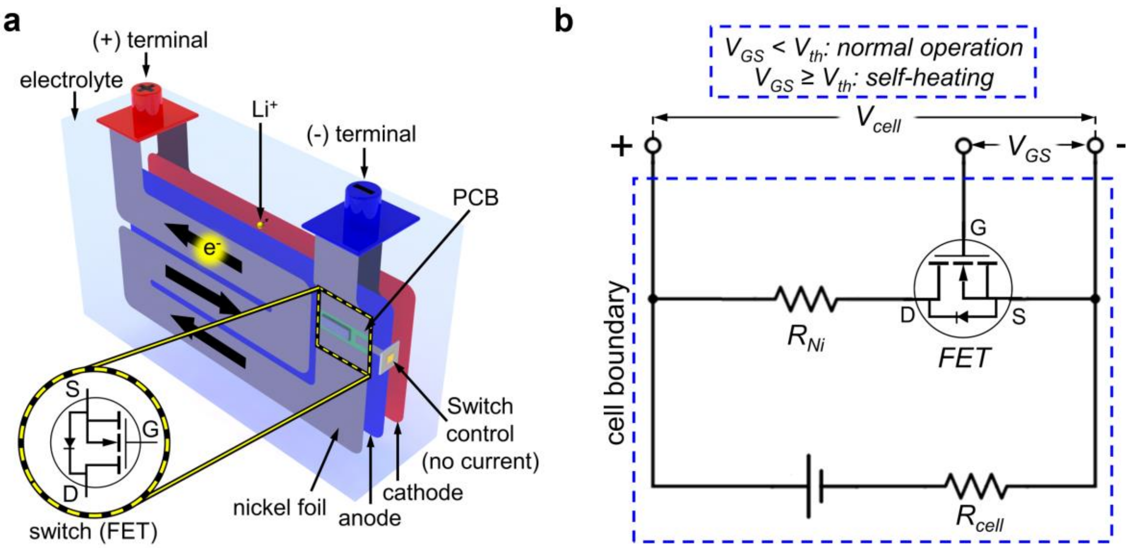

Supplementary Fig. 2 | iSHB schematics. a, Graphical and **b**, electrical iSHB schematics. An n-type, enhancement mode field effect transistor (FET) is mounted on a thin printed circuit board (PCB), embedded in-plane/in series with a Ni heating foil, and sandwiched between the electrodes to achieve a heating and control circuit that mutually heats the battery materials and cools the transistor. The gate voltage (V_{GS}) is controlled with a small electrical contact outside the cell. Raising V_{GS} above the threshold voltage of the FET (V_{th}) turns on the switch, activating heating. When V_{GS} is held below V_{th}, heating is inactive, leaving the electrochemical cell to function with "normal" operation.

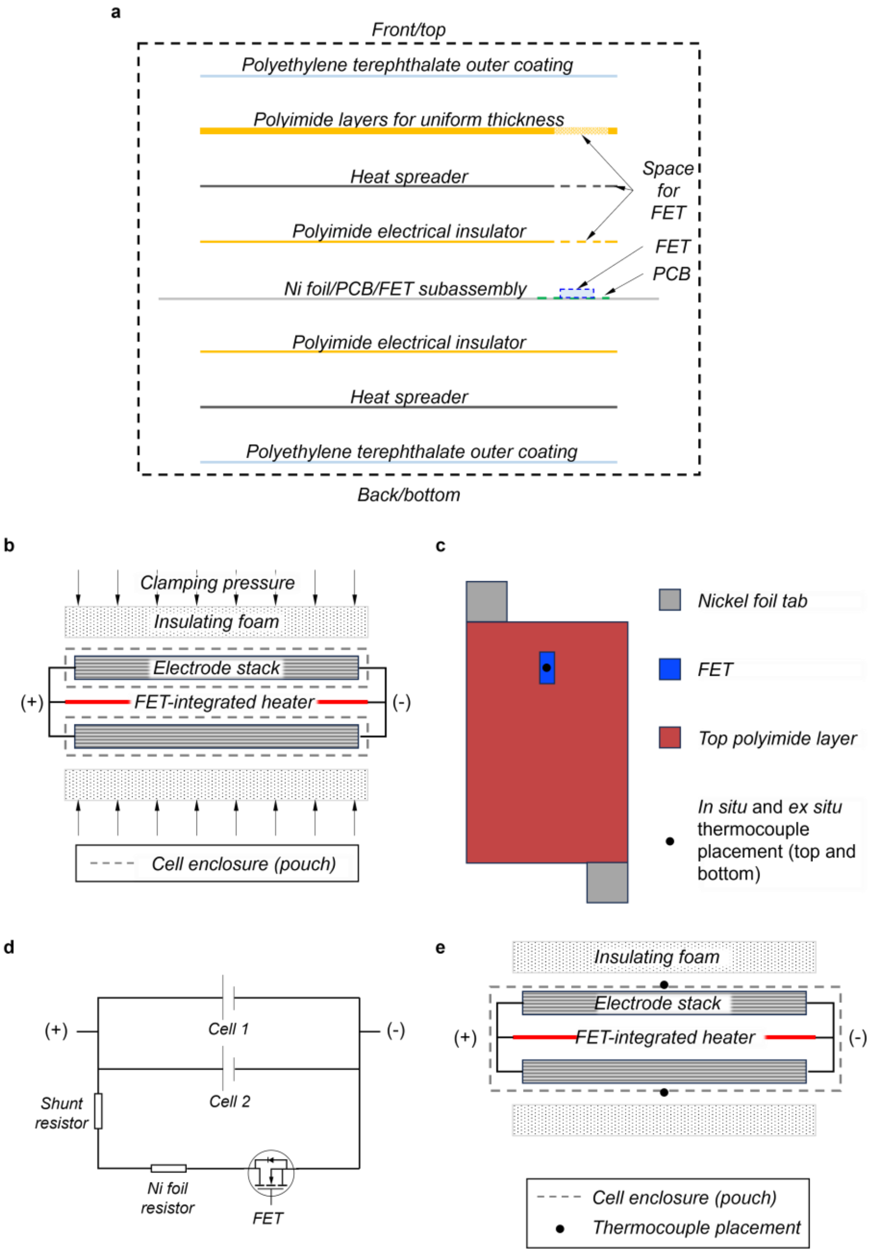

Supplementary Fig. 3 | iSHB heating sheet structure and experimental configurations. (**a**) **Stack**-up of iSHB heating sheet constituent materials (not to scale). The parylene-C conformal coating, which covers all outer surfaces (inside the polyethylene terephthalate) including the field effect transistor (FET) and exposed printed circuit board (PCB), is not shown. Mock iSHB (**b**) experimental stack up, (**c**) thermocouple placement, and (**d**) electrical schematic. The mock iSHB used for *in situ* experiments utilized two half thickness cells inside their own enclosure wired in parallel with the FET-integrated heater. The cells sandwiched the iSHB heating element, which was instrumented with thermocouple on the top and bottom in the location of the FET. Insulating foam and external clamping pressure were applied to ensure representative interfacial thermal contact between the heating sheet and the cells. (**e**) Fully integrated iSHB: a single cell with the FET-integrated heater embedded inside the cell during electrode stacking and thermocouples placed on the cell skin on both top and bottom.

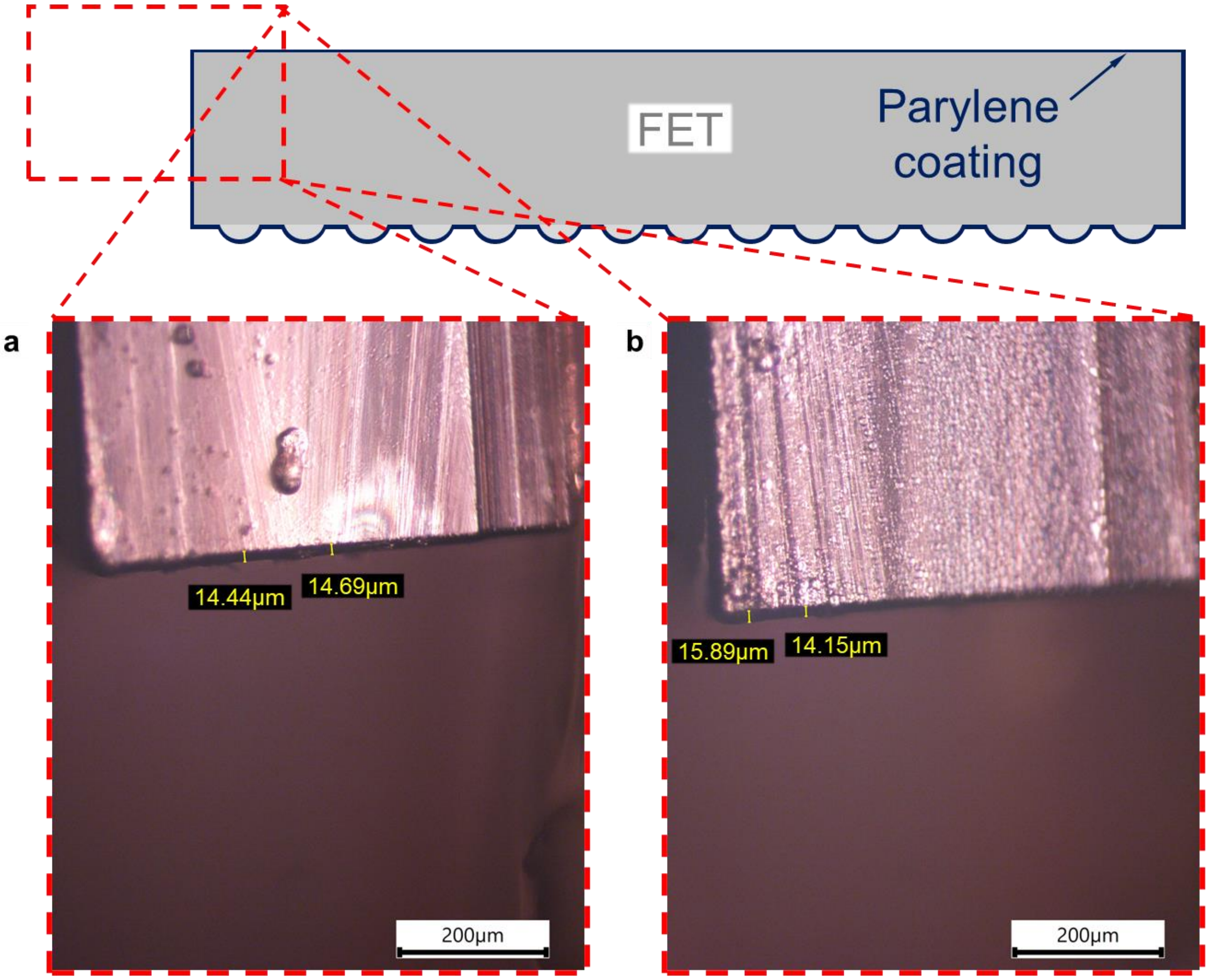

Supplementary Fig. 4 | Optical microscopy of parylene coating. a, and **b,** Images of the corner of two field effect transistors (FETs) coated with parylene-C obtained at 50x magnification with an optical microscope. Calibrated measurements indicate the parylene coating thickness is ~15 µm thick. The graphic above both panels indicates the region of the FET that was imaged.

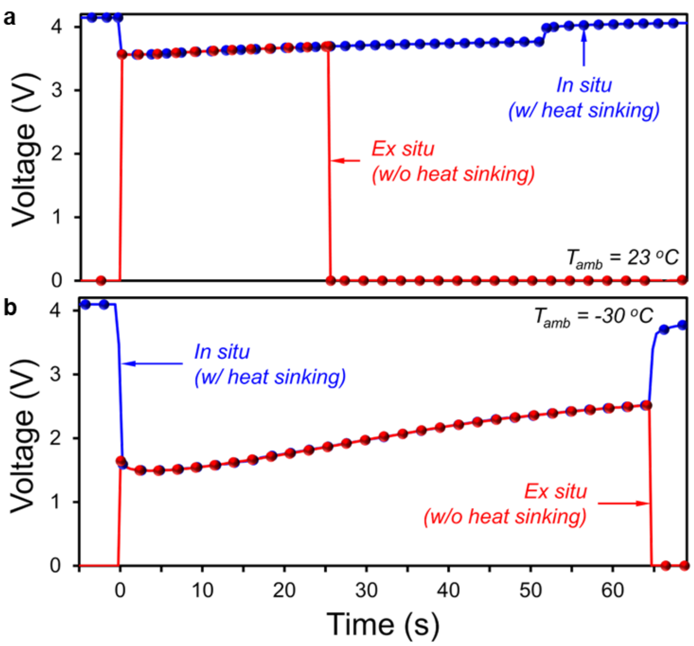

Supplementary Fig. 5 | Voltage evolution during *in situ* and *ex situ* thermal characterization. For **a**, room temperature and **b**, -30 °C environments: Heating element voltage evolution. The voltage profiles observed during *in situ* heating experiments were imposed on the heating foil to apply comparable levels of heating power for comparison of thermal performance. T_{amb} represents the temperature of the ambient environment.

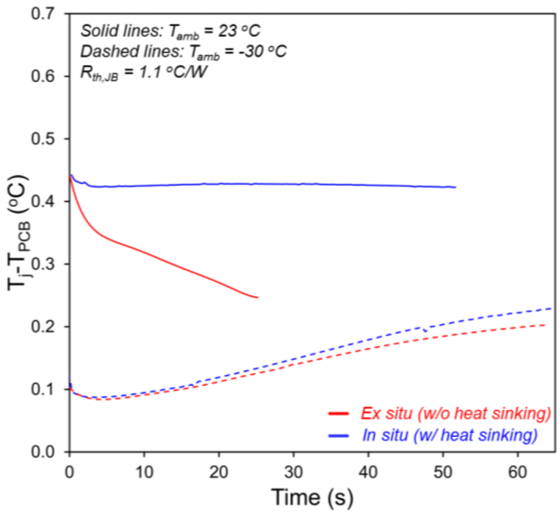

Supplementary Fig. 6 | Junction temperature evolution during heating. Temperature difference between the FET junction (T_j) and printed circuit board (T_{PCB}) during *ex situ* and *in situ* heating tests. The junction temperature is estimated based on $T_j = T_{PCB} + (I^2 R_{DS(on)})(R_{th,JB})$ with the FET resistance ($R_{DS(on)}$) evaluated at the PCB temperature, the current (I) and the junction-to-board thermal resistance ($R_{th,JB}$) of 1.1 °C/W as provided by the FET manufacturer (EPC Technologies).

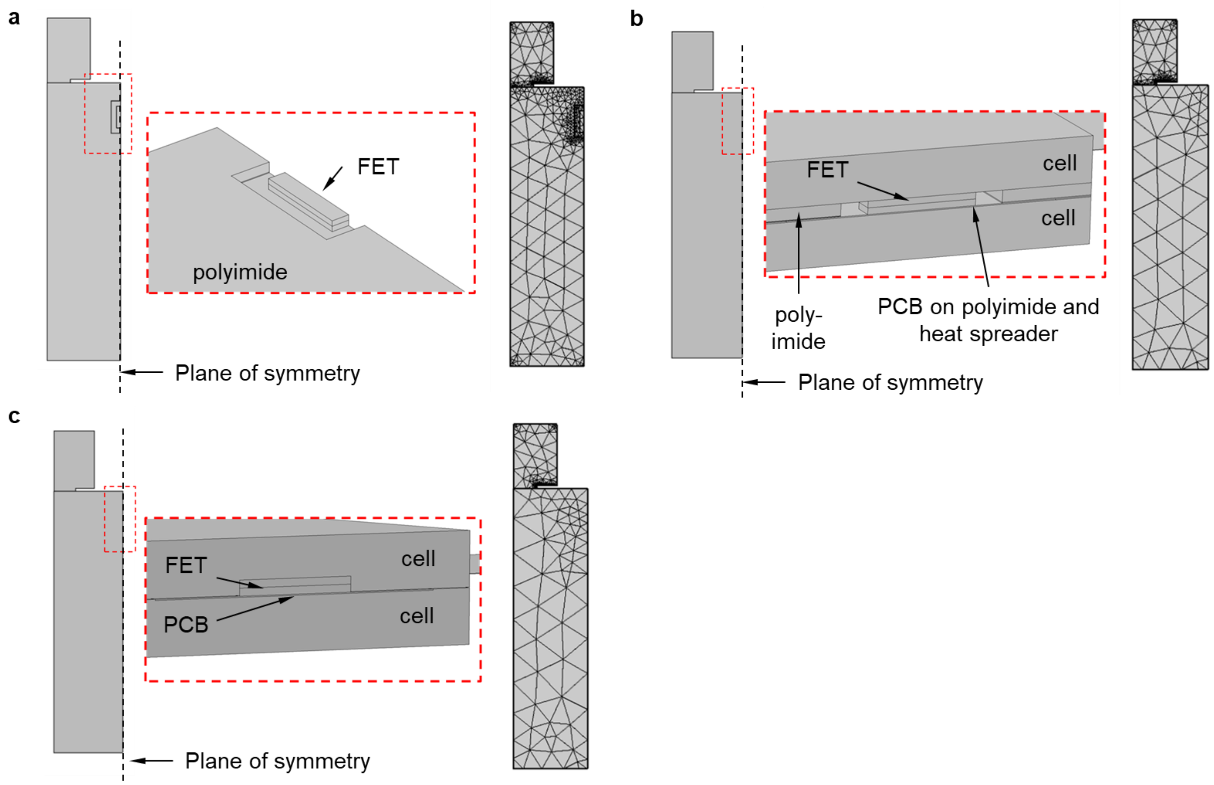

Supplementary Fig. 7 | Model geometry and mesh for numerical simulations of heating element and iSHB heating. a, Heating element, **b**, prototype iSHB, and **c**, thermally-optimized iSHB. The plane of symmetry (vertical, dashed line in each panel) was implemented to minimize computational expense. Each panel contains the top view of the three-dimensional model, a magnified view of the FET and surrounding region, and the top view of the three-dimensional model with the applied mesh (left to right).

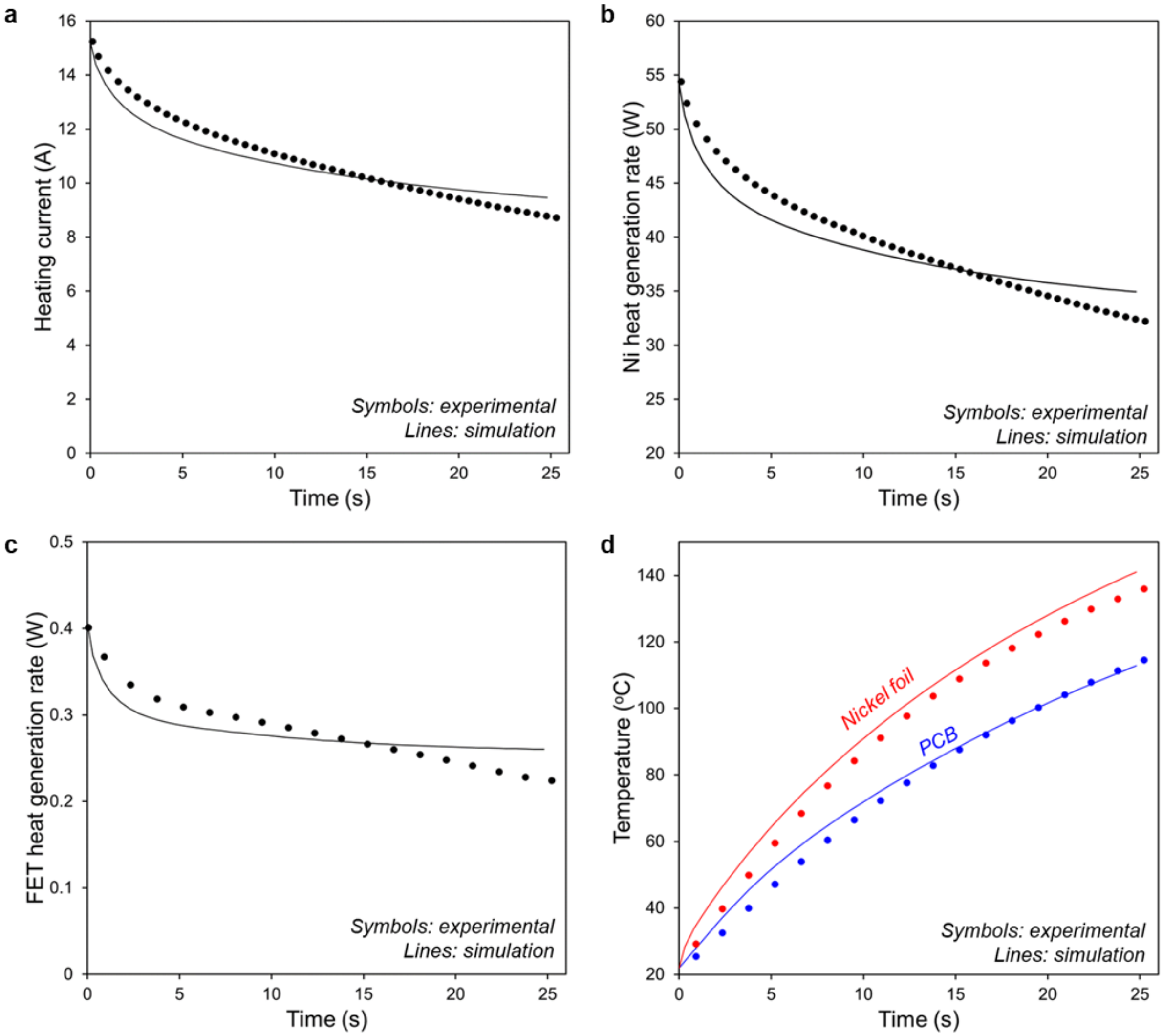

Supplementary Fig. 8 | Validation of simulation of *ex situ* heating of prototype heating element from room temperature. a, **b**, **c**, and **d**: Experimental and simulation results vs. time for heating current, Ni foil heat generation, FET heat generation, and key temperatures, respectively.

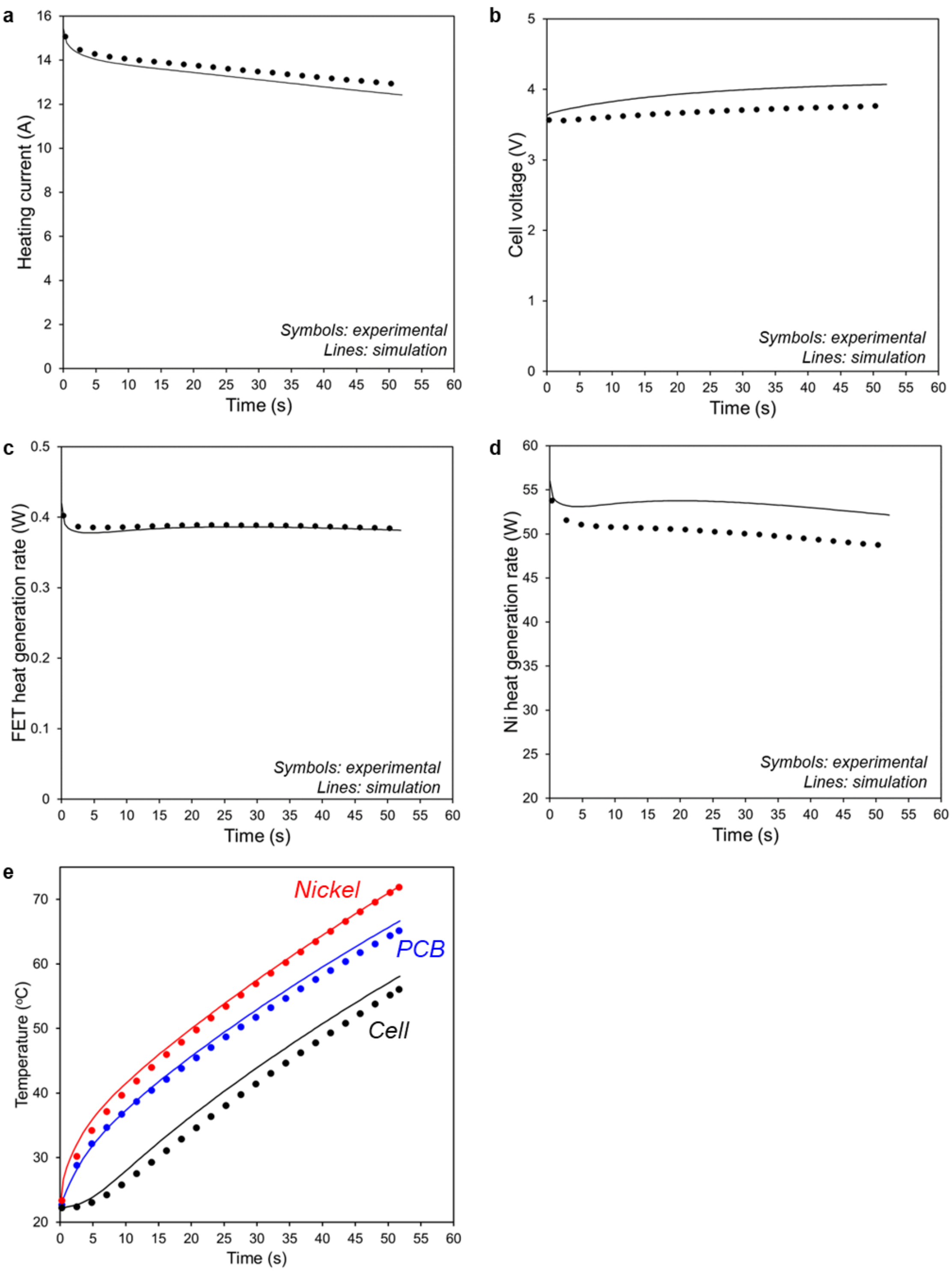

Supplementary Fig. 9 | Validation of simulation of *in situ* heating of prototype iSHB from room temperature. a, **b**, **c**, **d**, and **e**: Experimental and simulation results vs. time for heating current, cell voltage, FET heat generation, Ni foil heat generation, and key temperatures, respectively.

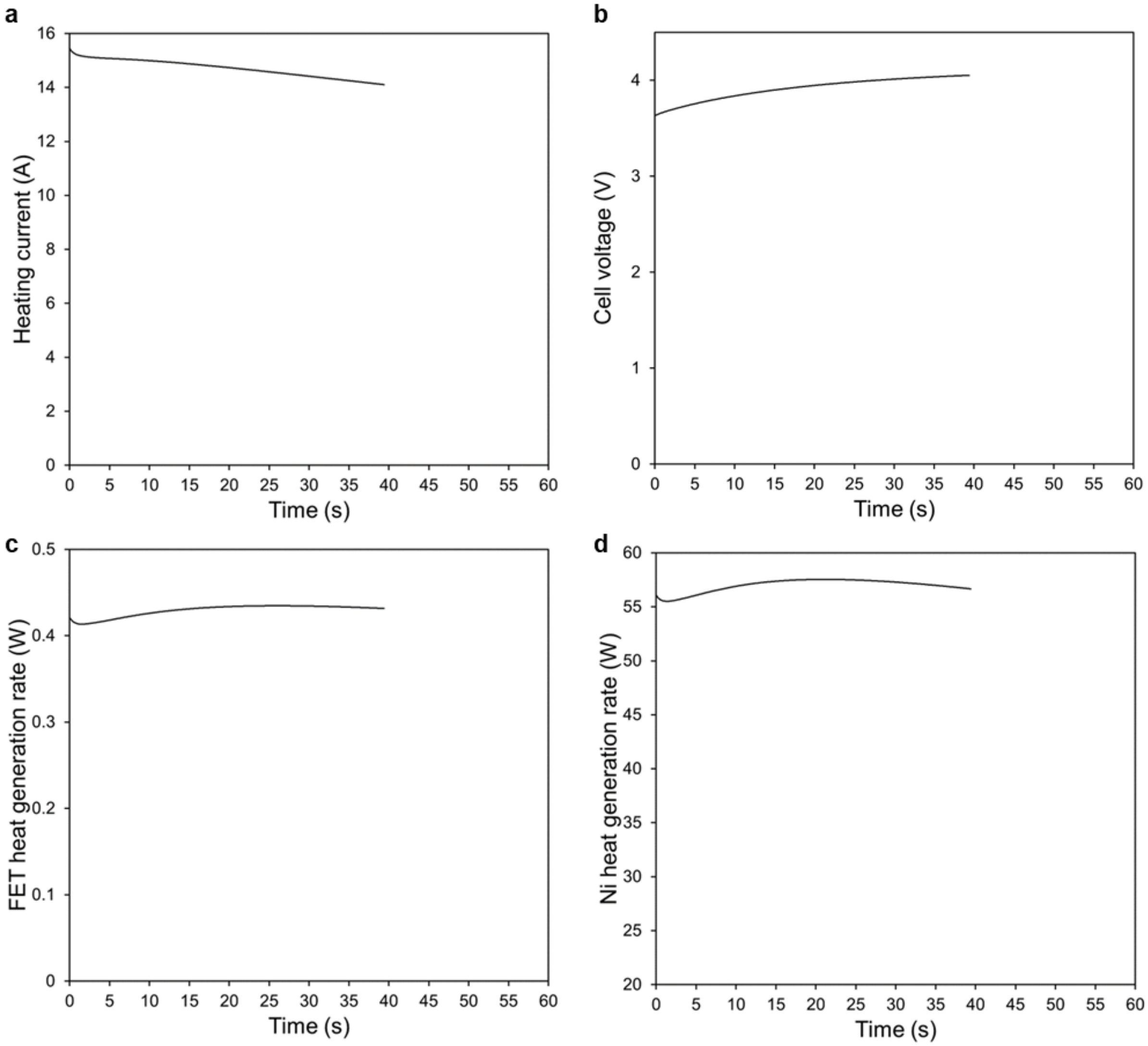

Supplementary Fig. 10 | Simulation of *in situ* heating of thermally optimized iSHB from room temperature. a, **b**, **c**, and **d**: Simulation results vs. time for heating current, cell voltage, FET heat generation, and Ni foil heat generation, respectively. The evolution of key temperatures is provided in Fig. 2d.

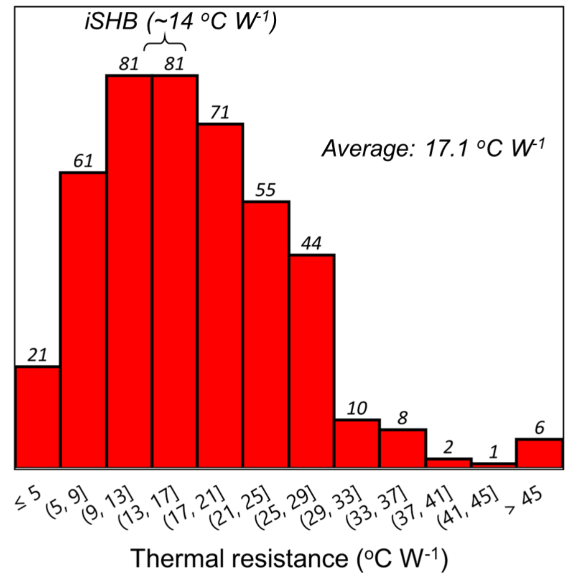

Supplementary Fig. 11 | Survey of common transistor heat sinks. Histogram all 441 unique off-the-shelf heat sinks corresponding to the survey data presented in Fig. 2f.

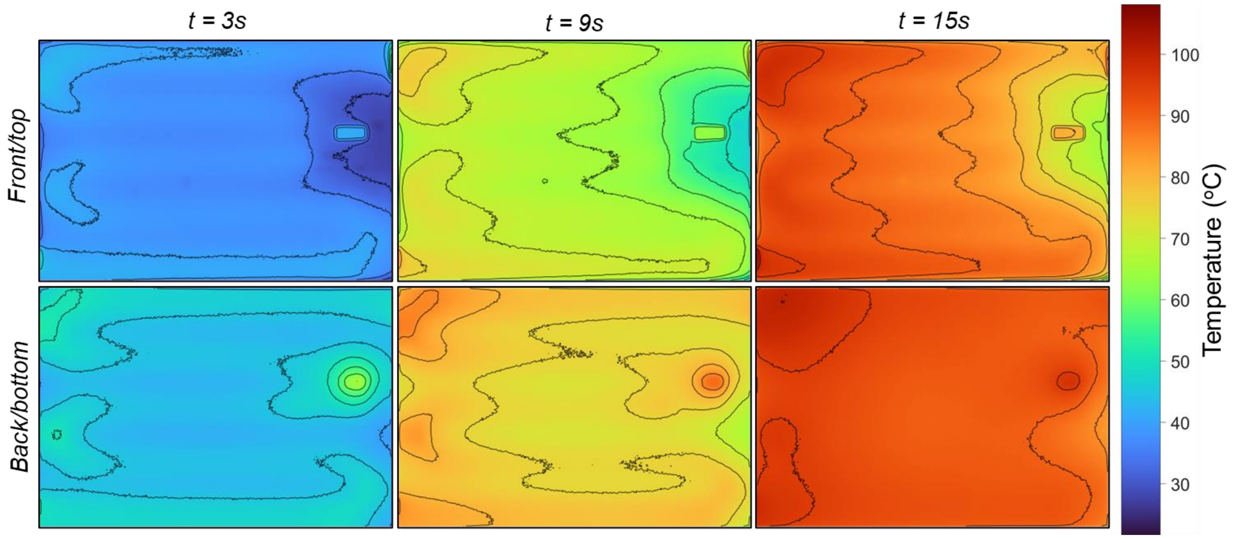

Supplementary Fig. 12 | Contoured thermographs during *ex situ* heating from room temperature. Thermographs from IR thermography of the front/top and back/bottom of the iSHB heating element at 3, 9, and 15 seconds, as labeled. The contour lines correspond to a threshold temperature difference of 5 °C. The region of interest corresponds to that defined in Supplementary Movie 1.

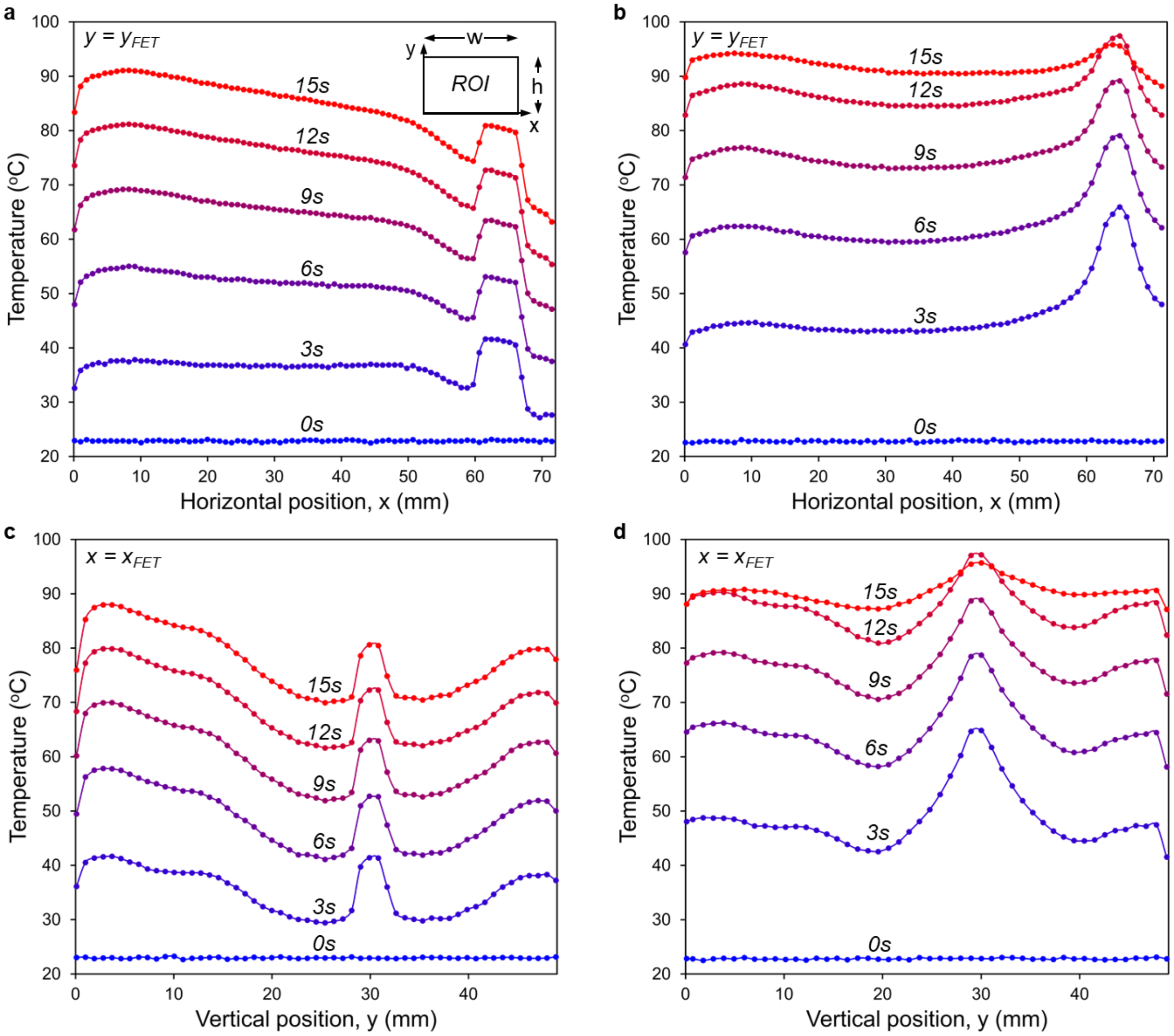

Supplementary Fig. 13 | Temperature distributions during *ex situ* heating. As measured with infrared (IR) thermography in RT ambient: Temperature along the horizontal line that intersects the center of the FET on the front/top (**a**) and back/bottom (**b**) of the heating sheet and temperature along the vertical line that intersects the center of the FET on the front/top (**c**) and back/bottom (**d**) of the heating sheet for the regions of interest (ROI) as defined in Supplementary Movie 1. The inset in a defines the coordinate system applied for the ROI in this figure and in Supplementary Movie 1. While the FET/PCB generates a mild, local hotspot, the heating sheet maintains moderately uniform temperature during *ex situ* heating, supporting even better uniformity *in situ*.

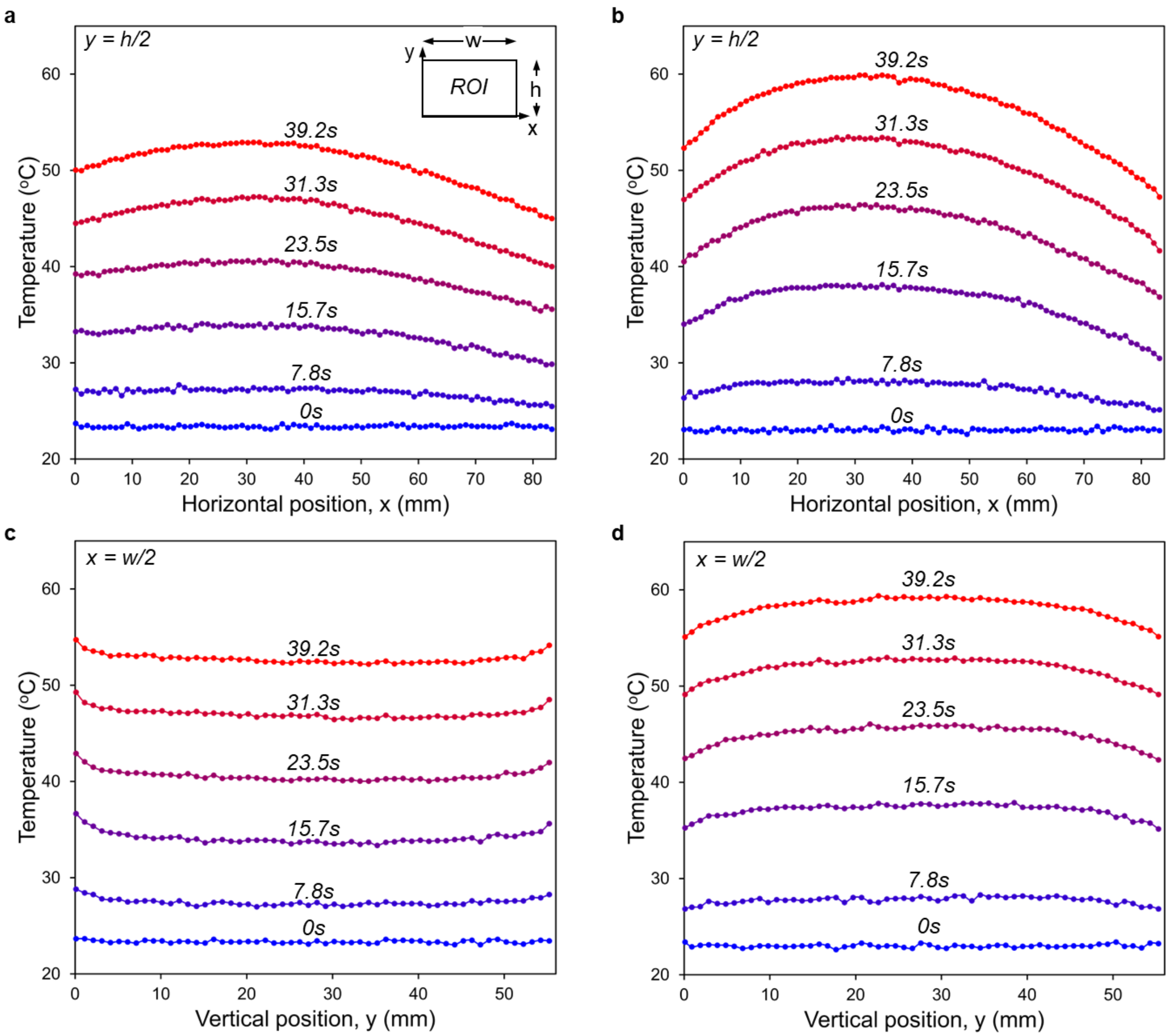

Supplementary Fig. 14 | iSHB surface temperature distributions during heating from room temperature. As measured with infrared (IR) thermography: Temperature along the horizontal centerline on the front/top (**a**) and back/bottom (**b**) of the iSHB surface and temperature along the vertical centerline on the front/top (**c**) and back/bottom (**d**) of the iSHB surface for the regions of interest (ROI) defined in Supplementary Movie 2. The inset in a defines the coordinate system applied for the ROI in this figure and in Supplementary Movie 2.

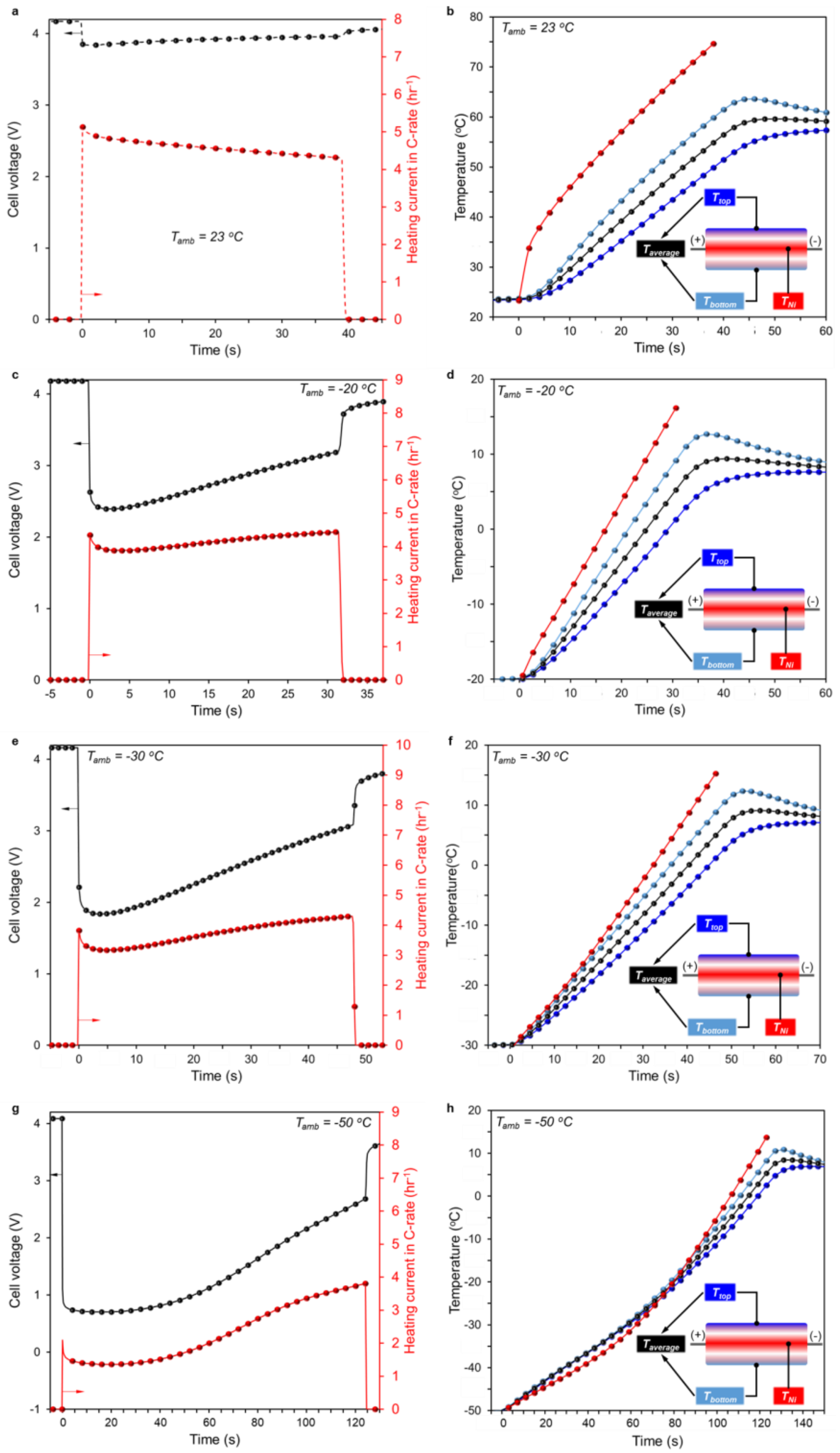

Supplementary Fig. 15 | iSHB cell response during activation. Cell voltage and heating current evolution during heating from 23 to 60 °C (**a**), and heating to 5 °C from -20 (**c**), -30 (**e**), and -50 °C (**g**). Evolutions of nickel foil (T_{Ni}), top surface (T_{top}), bottom surface (T_{top}), and average surface temperature ($T_{average}$) during heating from 23 °C to 60 °C (**b**) and self-heating to 5 °C from -20 (**d**), -30 (**f**), and -50 °C (**h**). The insets in **b**, **d**, **f**, and **h** illustrate the location of thermocouples (T_{top} and T_{bottom}) and the heating element (T_{Ni}), where the average data plotted consists of only T_{top} and T_{bottom}.

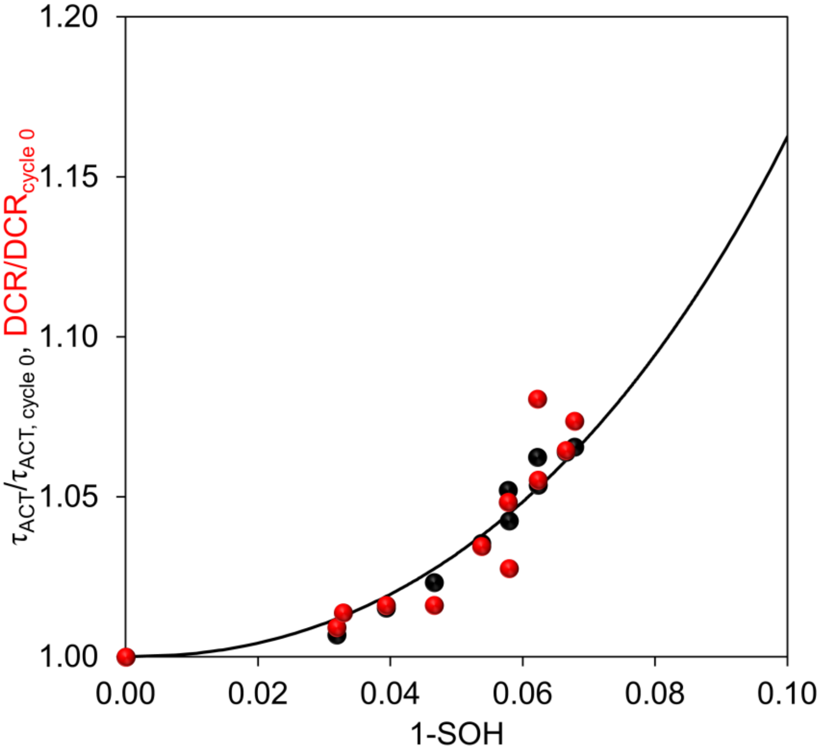

Supplementary Fig. 16 | Impact of repetitive thermal activation on heating time and direct current resistance. Heating time and direct current resistance (DCR) vs. fractional capacity loss shown as 1-state of health (SOH). The DCR data is calculated from the first 30 seconds of discharge during each reference performance test throughout the repetitive thermal activation experiment. A close correlation between the trend in normalized heating time and normalized DCR is observed.

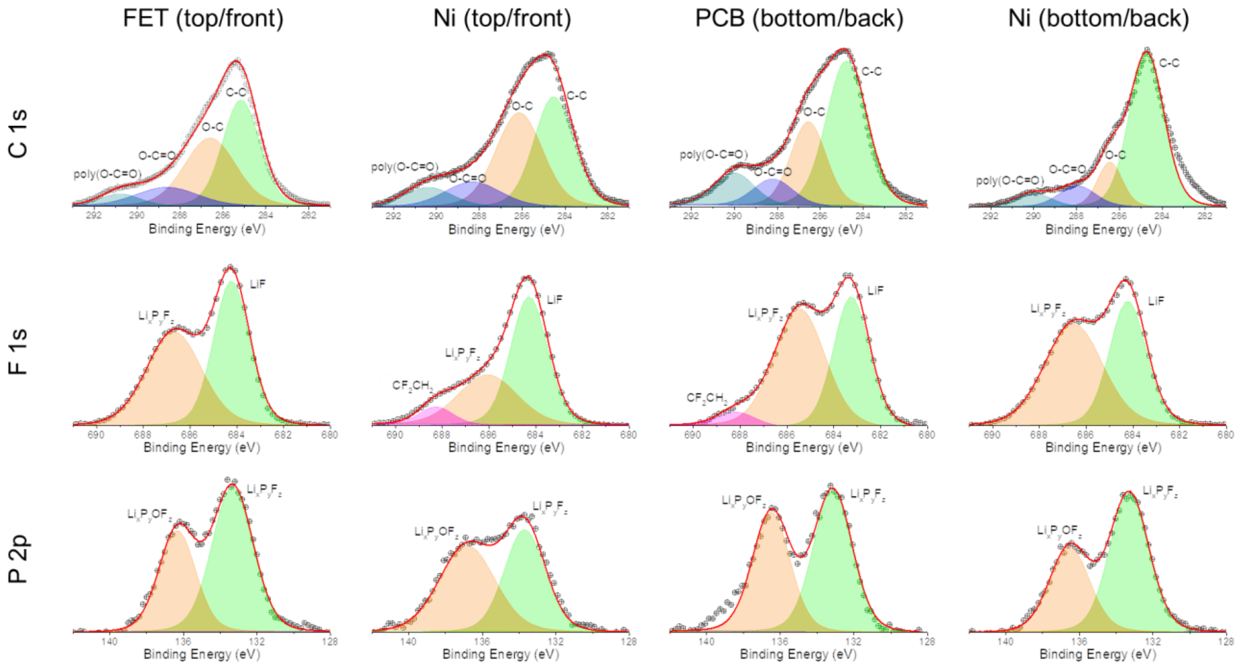

Supplementary Fig. 17 | Surface chemistry of thermally cycled anodes. X-ray photoelectron spectra (XPS) from scans of the graphite anodes adjacent to the heating sheet after 1,000 heating from 30°C to ~53°C in a room temperature ambient condition (~23 °C). The four samples were harvested from the anodes adjacent to both sides of the heating sheet (top/front and back/bottom) at regions over the FET/PCB and Ni foil approximately equidistant from the FET and the anode edge. The results show no substantial abnormalities or discrepancies among the four regions, indicating that the temperature is sufficiently uniform during heating and/or the time exposed to heating is sufficiently short to avoid any deleterious non-uniform aging effects.

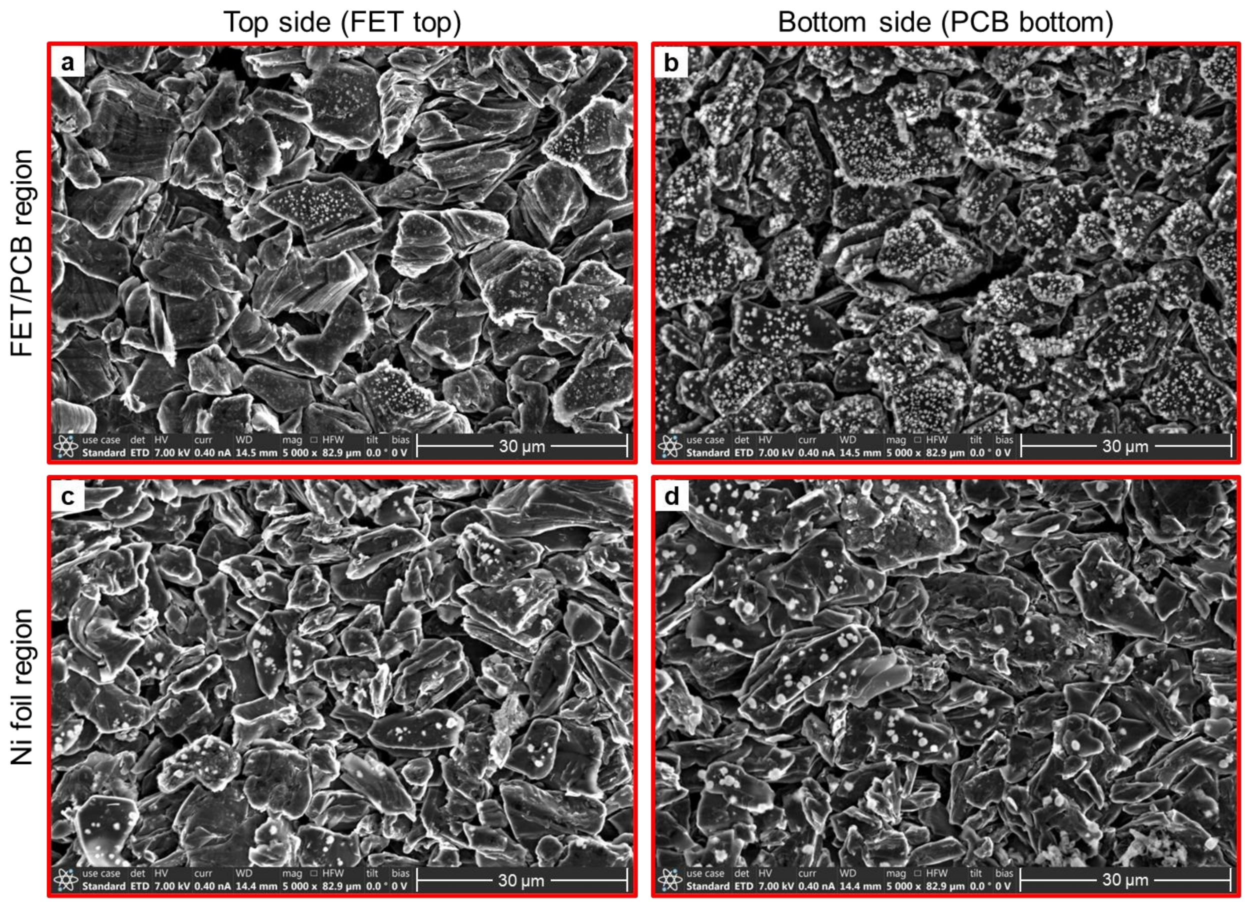

Supplementary Fig. 18 | Anode SEM micrographs after repetitive heating aging. SEM micrographs of the graphite anodes adjacent to the heating sheet after 1,000 heating from 30°C to ~53°C in a room temperature ambient condition (~22 ^{o}C). (**a** and **b**) were harvested from the region near the FET-PCB assembly on the top of the FET and bottom of the PCB, respectively. (**c** and **d**) were harvested from the Ni foil region approximately equidistant from the FET and the anode edge on the top of the FET and bottom of the PCB, respectively. The white spots on the graphite particles are residual salt that did not dissolve during washing with dimethyl carbonate solvent. No notable differences appear in the porous structure or graphite particles.

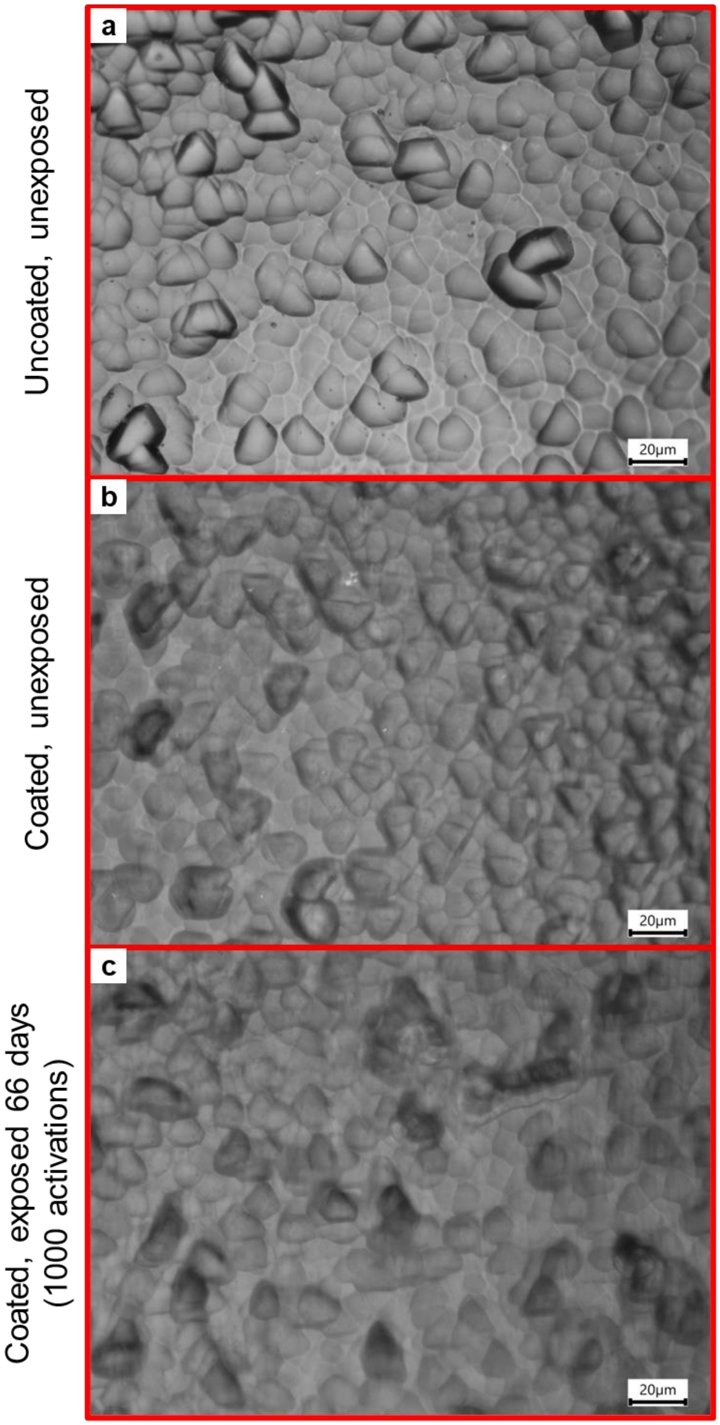

Supplementary Fig. 19 | Optical microscopy of top of FET surface. Images of glass passivation layer on the top surface of FET: **a**, uncoated and **b**, parylene-C coated without exposure to electrolyte and **c**, parylene-C coated with exposure to electrolyte for 66 days during which ~1,000 heating cycles from 30 ^{o}C to ~53 ^{o}C were performed. The parylene-C coated samples show no distinct difference, whether exposed to electrolyte (with ~1,000 activations) or not.

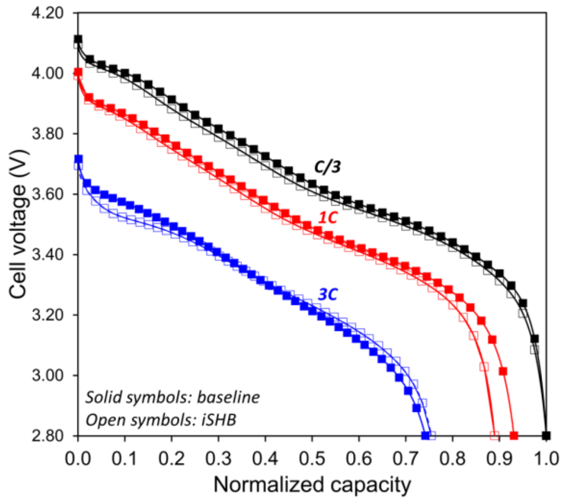

Supplementary Fig. 20 | Baseline LIB and iSHB rate performance. Voltage vs. capacity normalized to that at C/3 rate for C/3, 1C, and 3C rates for the half-thickness, conventional LIB and iSHB.

Supplementary Tables

Supplementary Table 1 | Cell design: conventional LIB and iSHB.

Cell Component	Parameter	Baseline LIB	Prototype iSHB	Optimized iSHB
Cathode	AM capacity (mAh g^{-1}) C/10		194	
	N/P ratio		1.1	
	Single-sided electrode thickness (μm)		53.8	
	AM mass loading, single-sided (mg cm^{-2})		16.8	
	Areal capacity (mAh/cm^{-2})		3.26	
Anode	Single-sided electrode thickness (μm)		75	
	AM mass loading, single-sided (mg cm^{-2})		10.8	
Current collector	Al for cathode (μm)		13	
	Cu for anode (μm)		8	
Separator	Thickness (μm)		25	
Heating sheet	Mass (g)	0	4.94	1.28
	Thickness (μm)	0	836	75
	Volume x 10^3 (L)	0	4.57	0.41
Full cell	Cathode footprint area (cm^2)		40.56	
	Double-sided cathode layers		12	
	Cell capacity (Ah)		3.17	
	Nominal discharge voltage (V)		3.7	
	Cell mass (g)	54.4	61.9	55.7
	Cell volume (mL)	20.6	27.0	21.0
	Specific energy (Wh kg^{-1})	216	189	211
	Energy density (Wh L^{-1})	570	434	559

Active material (AM)
See Methods for specific energy and energy density calculation details.

Supplementary Table 2 | Prototype and Optimized iSHB heating sheet and cell mass and specific heat.

	Component	Mass (g)	Mass fraction	Specific heat (J kg^{-1} K^{-1})	Specific heat contribution (J kg^{-1} K^{-1})
**Heating sheet**	Ni foil	0.70	0.142	460	65.1
	FET	0.03	0.005	548	2.8
	Polyimide insulator	0.66	0.133	1090	145.5
	Heat spreader	0.22	0.044	708	31.2
	Polyimide film	2.78	0.563	1090	613.6
	PCB	0.01	0.002	390	0.8
	PET film	0.36	0.073	1250	91.2
	Parylene-C	0.19	0.038	711	27.0
	**Total**	**4.94**	**1.000**		**977**
**Prototype iSHB**	Heating sheet	4.94	0.080	977	78.1
	Cathode coating	16.73	0.270	634	171.5
	Al foil	1.74	0.028	890	25.1
	Anode coating	13.46	0.218	725	157.7
	Cu foil	4.53	0.073	385	28.2
	Separator	1.62	0.026	1800	47.1
	Electrolyte	7.90	0.128	1690	215.8
	Ni/Cu tabs	5.00	0.081	400	32.3
	Al tabs	1.95	0.032	890	28.1
	Enclosure	4.00	0.065	1149	74.3
	**Total**	**61.87**	**1.000**		**858**
**Optimized Heating sheet**	Ni foil	0.70	0.546	460	251.0
	FET	0.03	0.019	548	10.7
	Polyimide insulator	0.00	0.000	1090	0.0
	Heat spreader	0.00	0.000	708	0.0
	Polyimide film	0.00	0.000	1090	0.0
	PCB	0.01	0.008	390	2.9
	PET film	0.36	0.281	1250	351.3
	Parylene-C	0.19	0.146	711	104.0
	**Total**	**1.28**	**1.000**		**720**
**Optimized iSHB**	Heating sheet	1.28	0.023	720	16.6
	Cathode coating	16.73	0.300	634	190.5
	Al foil	1.74	0.031	890	27.9
	Anode coating	13.46	0.242	725	175.2
	Cu foil	4.53	0.081	385	31.3
	Separator	1.62	0.029	1800	52.3
	Electrolyte	7.90	0.142	1690	239.7
	Ni/Cu tabs	2.50	0.045	400	18.0
	Al tabs	1.95	0.035	890	31.2
	Enclosure	4.00	0.072	1149	82.5
	**Total**	**55.7**	**1.000**		**865**

Supplementary Table 3 | Material properties for numerical simulations.

Component/material	Density ($kg\ m^{-3}$)	Specific heat ($J\ kg^{-1}\ K^{-1}$)	Thermal conductivity ($W\ m^{-1}\ K^{-1}$)
PCB (copper, polyimide, gold plating, solder mask)	10,000	1,600	100
Nickel	8,902	444	80
FET	6,150	548	250
Polyimide (w/ adhesive)	1,420	1,100	0.06
Heat spreader (w/ adhesive)	2,265	850	100
LIB Half cell	2,200	1,300	0.8
