# Supplementary material for: Battery electronification: intracell actuation and thermal management
Source: Nat Commun. 2024 Jun 25;15:5373. doi: 10.1038/s41467-024-49389-5 (PMC11199607; doi:10.1038/s41467-024-49389-5)
Supplement: Supplementary file 3 — Description of Additional Supplementary Files [file 41467_2024_49389_MOESM3_ESM.pdf]

Description of Additional Supplementary Files

Supplementary Movie 1: Infrared thermography of iSHB heating sheet during ex situ heating in room temperature ambient. The evolution of thermographs are shown at the top for the front/top and back/bottom of the heating sheet as labeled. Surface plots of the evolution of temperature distribution corresponding to the region of interest (ROI) indicated by the dashed line in corresponding thermographs are shown at the bottom. The FET and PCB regions used for average FET or PCB temperature are denoted by the solid rectangular region in the corresponding thermographs. The domain for the average temperature is the entire ROI. The origin for the horizontal and vertical positions lies at the bottom left corner of the ROI.

Supplementary Movie 2: Infrared thermography of iSHB surface during heating in room temperature ambient. The evolution of thermographs are shown at the top for the front/top and back/bottom of the iSHB as labeled. Surface plots of the evolution of temperature distribution corresponding to the region of interest (ROI) indicated by the dashed line in the corresponding thermographs are shown at the bottom. The origin for the horizontal and vertical positions lies at the bottom left corner of the ROI. The domain for the average temperature is the entire ROI.
